# Phosphatidylthreonine is a procoagulant lipid detected in human blood and elevated in coronary artery disease

**DOI:** 10.1016/j.jlr.2023.100484

**Published:** 2023-12-14

**Authors:** Ali A. Hajeyah, Majd B. Protty, Divyani Paul, Daniela Costa, Nader Omidvar, Bethan Morgan, Yugo Iwasaki, Beth McGill, P. Vincent Jenkins, Zaheer Yousef, Keith Allen-Redpath, Shin Soyama, Anirban Choudhury, Rito Mitra, Parveen Yaqoob, James H. Morrissey, Peter W. Collins, Valerie B. O'Donnell

**Affiliations:** 1 Systems Immunity Research Institute and Division of Infection and Immunity, Cardiff University, Cardiff, United Kingdom; 2 Department of Biological Sciences, Kuwait University, Safat, Kuwait; 3 Department of Biological Chemistry, University of Michigan, Ann Arbor, MI, USA; 4 College of Bioscience and Biotechnology, Chubu University, Kasugai, Japan; 5 University Hospital of Wales, Cardiff, United Kingdom; 6 Department of Food and Nutritional Sciences, University of Reading, Reading, United Kingdom; 7 Morriston Cardiac Centre, Swansea, United Kingdom

**Keywords:** phospholipids, phospholipids/metabolism, phospholipids/biosynthesis, vascular biology, platelets, phosphatidylthreonine, blood coagulation, coronary artery disease

## Abstract

Aminophospholipids (aPL) such as phosphatidylserine are essential for supporting the activity of coagulation factors, circulating platelets, and blood cells. Phosphatidylthreonine (PT) is an aminophospholipid previously reported in eukaryotic parasites and animal cell cultures, but not yet in human tissues. Here, we evaluated whether PT is present in blood cells and characterized its ability to support coagulation. Several PT molecular species were detected in human blood, washed platelets, extracellular vesicles, and isolated leukocytes from healthy volunteers using liquid chromatography–tandem mass spectrometry. The ability of PT to support coagulation was demonstrated in vitro using biochemical and biophysical assays. In liposomes, PT supported prothrombinase activity in the presence and absence of phosphatidylserine. PT nanodiscs strongly bound FVa and lactadherin (nM affinity) but poorly bound prothrombin and FX, suggesting that PT supports prothrombinase through recruitment of FVa. PT liposomes bearing tissue factor poorly generated thrombin in platelet poor plasma, indicating that PT poorly supports extrinsic tenase activity. On platelet activation, PT is externalized and partially metabolized. Last, PT was significantly higher in platelets and extracellular vesicle from patients with coronary artery disease than in healthy controls. In summary, PT is present in human blood, binds FVa and lactadherin, supports coagulation in vitro through FVa binding, and is elevated in atherosclerotic vascular disease. Our studies reveal a new phospholipid subclass, that contributes to the procoagulant membrane, and may support thrombosis in patients at elevated risk.

Coagulation requires generation of an electronegative membrane on the surface of platelets, provided by aminophospholipids (aPL). Phosphatidylserine (PS) is required for coagulation reactions and phosphatidylethanolamine (PE) supports the action of PS (1, 2). aPL are enriched in the inner plasma membrane leaflet of mammalian cells, and their externalization following activation facilitates extracellular vesicle (EV) shedding (e.g., by platelets) and supports coagulation by providing a surface for binding of coagulation factors (3). This binding is mediated by either γ-carboxyglutamate-rich (Gla) domains, found in FII, FVII, FIX, and FX, or discoidin (C2-like) domains in FVa and FVIIIa (4, 5, 6). Blood coagulation is initiated on surfaces of tissue factor (TF)-expressing cells where TF forms a complex with FVII, leading to its activation to FVIIa (7). The TF:FVIIa complex (extrinsic tenase) activates FX to FXa, which efficiently converts prothrombin (FII) into thrombin (FIIa) after its association with FVa and formation of the prothrombinase complex (FXa:FVa).

Increased thrombotic risk is a central feature of all forms of cardiovascular disease. While aPL have been recognized to provide the procoagulant membrane for decades, whether alterations to this surface contribute to elevated thrombotic risk is unclear. Aside from this the role of phospholipids in general, in cardiovascular disease is an area of increasing interest. Oxidized phospholipids either formed by cells or nonenzymatically are an emerging area of special interest since they are both procoagulant and pro-inflammatory (8, 9), while lysophospholipids are increasingly recognized as mediators of vascular development and inflammation. Some lysophospholipids are elevated in acute coronary syndromes, while others are reduced in populations at increased cardiovascular disease risk (10, 11, 12, 13).

A structural analog of PS containing a threonine head group, phosphatidylthreonine (PT), was previously reported in animal tissues, mammalian cell cultures, bacteria, and parasitic protozoa, but not in human tissues (14, 15, 16, 17, 18, 19, 20, 21, 22, 23). PT is abundant in *Toxoplasma gondii* and synthesized by phosphatidylthreonine synthase (22). There, PT functions in calcium homeostasis and virulence, and deletion of the phosphatidylthreonine synthase gene impairs the parasite’s virulence by disrupting motility, egress, and invasion (22, 24). In animal cells, PT is proposed to be synthesized by PS synthase (PSS) enzymes through a base-exchange reaction (19). Notably, PSS1 is a housekeeping enzyme based on mRNA expression data and as such, we predict that PT may be present in most tissues, but this is not yet known (25, 26). PT is typically detected using electrospray ionization mass spectrometry in the negative ion mode with normal phase separation and neutral loss of the headgroup ion of 101 Da (20, 27). This is similar to PS, which exhibits a neutral loss of 87 Da instead.

Based on its structural similarity to PS, we hypothesized that PT may function in blood coagulation by interacting with coagulation factors. Up to now, whether PT is even present in blood has not been examined nor has it ever been considered in the context of coagulation or cardiovascular disease. To test this, we investigate the occurrence of PT in human blood, platelets, EVs, and leukocytes using liquid chromatography–tandem mass spectrometry (LC-MS/MS). Next, we study the effects of thrombin activation on platelet PT. Then, we investigate the ability of PT to bind coagulation factors and support coagulation in vitro using surface plasmon resonance and coagulation assays, including a prothrombinase assay, calibrated automated thrombinography (CAT), and an extrinsic tenase assay (28).

To address whether PT could also be associated with CAD, we measured PT in platelets and EVs from a cohort of coronary artery disease patients (29), to investigate whether the lipid is altered in line with increased thrombotic risk in vascular disease.

## Materials and methods

### Blood collection from volunteers, and platelet isolation and activation

Blood donations were approved by the Cardiff University School of Medicine Ethics Committee (16/02 - Study: 8). Blood was drawn and platelets isolated and activated as described in Supplementary Methods. Separately, blood was collected from a clinical cohort as described in Supplementary Methods. Participants were recruited from Cardiff University and Cardiff and Vale University Health Boards, as described (29). Ethical approval was from Health and Care Research Wales (HCRW, IRAS 243701; REC reference 18/YH/0502). Isolation of platelets, extracellular vesicles and leukocytes was undertaken as described in Supplementary Methods. The human studies conducted and reported herein abide by the Declaration of Helsinki.

### Lipid extraction and LC-MS/MS analysis from whole blood, platelets, leukocytes, and EV

Lipids were extracted using liquid:liquid phase methods, outlined in full in Supplementary Methods. Acid hydrolysis and LC-MS/MS analysis were conducted as described in Supplementary Methods.

### Clinical cohort sample acquisition and processing

Lipid extracts of platelets, EV, and leukocytes from the clinical cohort were generated as outlined in Supplementary Methods and stored at −80°C (29). Following LC-MS/MS analysis for a previous study (29), the stored extracts were retrieved and added to an equal volume of methanol containing SPLASH mix (PS 15:0/18:1-D7: 0.05 ng/μl, final conc), then analyzed using LC-MS/MS as described in Supplementary Methods for PT (supplemental Table S2). As IS was added post-extraction, amounts of PT are not quantified, and data is presented as relative levels, normalized across lipid species, or patient groups. The cohort comprised four groups: healthy controls (HC, n = 24), risk factors with no significant coronary artery disease (RF, n = 23), significant coronary artery disease but no acute coronary syndrome (CAD, n = 19), and acute coronary syndrome (ACS, n = 24). Details on the cohort were recently reported by Protty *et al.* (29) and full information is in Supplementary Methods.

### Preparation of liposomes by membrane extrusion

Liposomes were made using PC 18:0/18:0, PE 18:0/18:1(9Z), PS 18:0/18:1(9Z), and PT 18:0/18:1(9Z) in Buffer (A: 10 mM HEPES, 10 mM NaCl, pH 7.35, or B: 20 mM HEPES, 140 mM NaCl, pH 7.35), followed by membrane extrusion as described in Supplementary Methods.

### Turbidimetric calcium binding assay

Analysis was performed using a UVIKON 923 double beam UV/VIS spectrophotometer (Agilent Technologies, formerly BioTek Instruments, Vermont). Liposomes (1 mM total lipid in 200 μl buffer) were added to a plastic microcuvette and absorbance measured at 400 nm, using Buffer A (10 mM HEPES, 10 mM NaCl, pH 7.35) as reference blank. Samples were titrated with 2 mM CaCl_2_ (in Buffer A) and changes in absorbance at 400 nm were monitored. Titration consisted of 10 additions of 2 μl CaCl_2_ (2 mM). Each addition was followed by mixing (pipetting), 1 min incubation, then absorbance measured.

### Prothrombinase, CAT, and extrinsic tenase

Liposomes were tested for their ability to interact and activate coagulation factors using in vitro assays described in full in Supplementary Methods.

### Preparation of nanodiscs and surface plasmon resonance

Nanodiscs were prepared by self-assembly reactions followed by gel filtration as described previously, using various ratios of PC 16:0/18:1(9Z), PS 16:0/18:1(9Z) and PT 16:0/18:1(9Z) (30). Full details are in Supplementary Methods.

### Statistical analysis

Statistics were performed using SPSS Statistics (version 27, IBM Corp, New York) or Excel (version 2201, Microsoft Corporation, Washington). For cohort samples, significance was determined using the Kruskal-Wallis H-test. Lipids in platelets were analyzed using paired t-tests (two-tailed). Lipids undetected in more than 50% of samples were excluded from analysis. For the purposes of statistical comparison, zeros (undetected analyte values) were replaced with 50% of the lowest value in the dataset (A/IS value of the smallest chromatographic peak that passed validation criteria). For coagulation assays, statistical significance was determined using one-way ANOVA and post-hoc Tukey tests.

## Results

### Characterization of PT species in human blood using LC-MS/MS

**Fig. 1:**
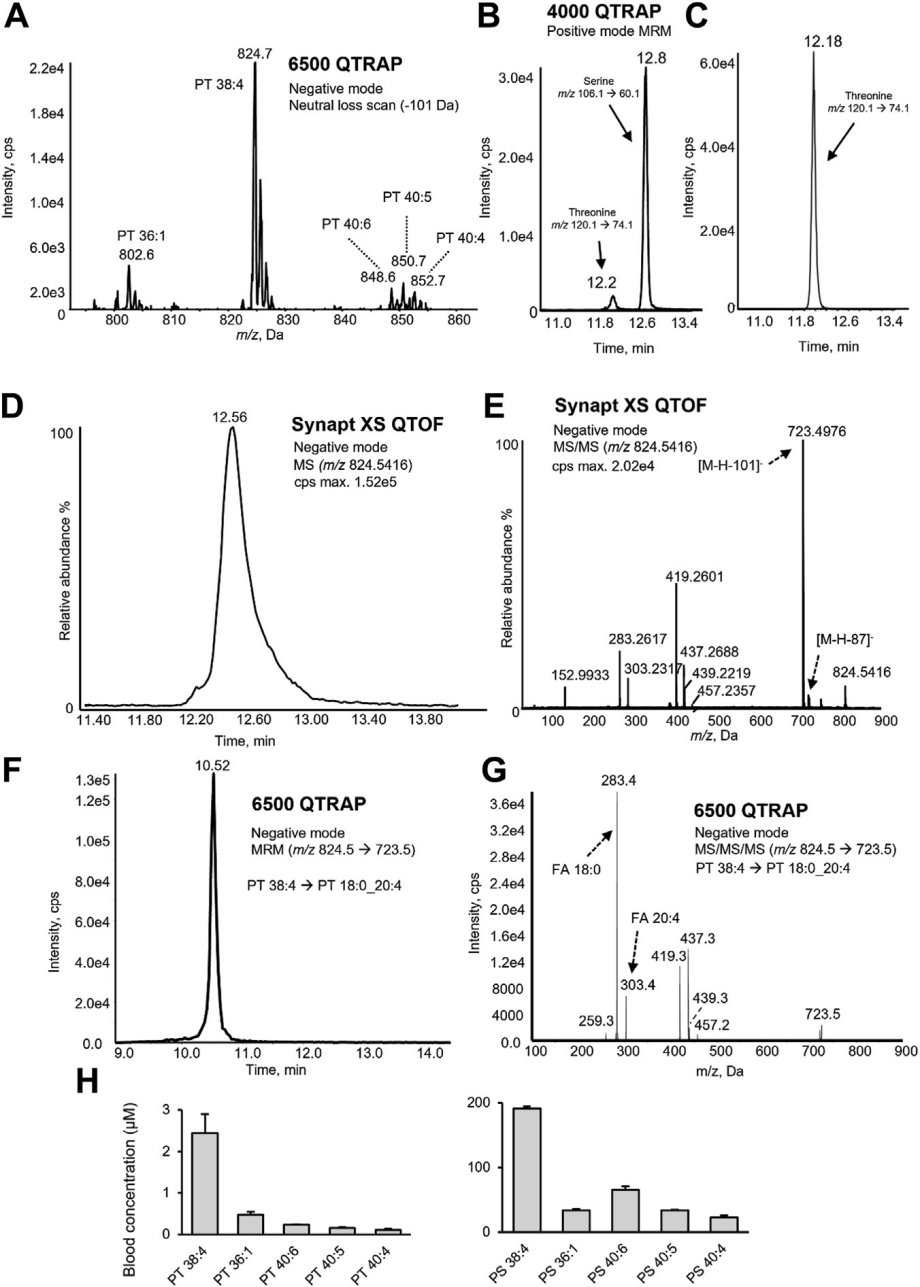
Whole blood contains several PT molecular species. A: Neutral loss-LC-MS/MS in negative ion mode scanning for loss of 101 Da in whole blood lipids shows several candidate ions. Lipids were extracted from whole blood and analyzed using neutral loss HILIC-LC-MS/MS as described in Supplementary Methods. Several putative PT molecules were detected. B: The PS/PT lipid fraction acid hydrolysate contains both serine and threonine. The PS/PT-rich lipid fraction was purified from whole blood lipids using HILIC chromatography and hydrolyzed using acid as outlined in Supplementary Methods; then the hydrolysate was analyzed using HILIC-LC-MS/MS. Detection of serine and threonine in the acid hydrolysate is shown. C: Detection of threonine in the acid hydrolysate of the PT standard. A PT 16:0/18:1(9Z) standard was hydrolyzed and analyzed using HILIC-LC-MS-MS. D: HILIC chromatography of PT 38:4 using LC-MS. Whole blood lipid extracts were analyzed using high resolution LC-MS for PT 38:4 at *m/z* 824.5416. The peak shape and tails suggest the presence of coeluting species. E: High resolution MS^2^ of *m/z* 824.5416 acquired at 12.5 min demonstrates the FA composition of PT 38:4 and confirms the threonine headgroup. The scan shows head loss fragments from two isomeric species: PT 38:4 (neutral loss of 101 Da) and PS 39:4 (neutral loss of 87 Da). FA are also detected at *m/z* 303 (FA 20:4) and 283 (FA 18:0). F: HILIC chromatography of PT 38:4 using LC-MS/MS. Platelet lipids were analyzed using HILIC-LC-MS/MS, monitoring precursor ion to product ion transitions (*m/z* 824.5 → 723.5). G: MS^3^ scan of PT 38:4 in platelet lipid extract with *m/z* 824.5 as first precursor and *m/z* 723.5 as second precursor, demonstrating the FA composition of PT 38:4. The scan shows *m/z* 283 (FA 18:0) and *m/z* 303 (FA 20:4), identifying the structure as PT 18:0_20:4. H: Quantification of the five candidate PT ions and PS in whole blood using MRM. Lipid extracts were analyzed using HILIC-LC-MS/MS and quantified as described in Supplementary Methods. Data are presented as means ± SEM (n = 3 separate donors). LC-MS/MS, liquid chromatography–tandem mass spectrometry; PS, phosphatidylserine; PT, phosphatidylthreonine.

To detect PT in human blood, lipid extracts were analyzed, scanning for neutral loss of the headgroup ion 101 Da in negative ion mode. This revealed five potential PT ions from *m/z* 600 to 900 (Fig. 1A). The ions were next detected using multiple reaction monitoring (MRM), targeting precursor ion to product ion transitions: *m/z* [M-H]^−^ → [M-H-101]^−^. The proposed PT lipids eluted slightly earlier than their corresponding phosphatidylserine (PS) species in hydrophilic interaction chromatography (HILIC-LC), consistent with the hydrophobicity conferred by the extra methyl group in PT compared to PS (supplemental Fig. S1). Importantly, PT 36:1 from blood eluted at the same retention time as the PT 36:1 standard (supplemental Fig. S2).

To further characterize the head group, a fraction rich in PS and PT was isolated from whole blood lipid extracts using HILIC-LC, then hydrolyzed using acid, as in Supplementary Methods. Lipid extracts were first verified as free of serine or threonine (supplemental Figs. S3 and S4). Free serine and threonine were detected in the hydrolysate using HILIC-LC-MS/MS in positive ion mode (Fig. 1B). Retention times of serine and threonine in the hydrolysate were consistent with those of L-serine and L-threonine primary standards (supplemental Fig. S3). Additionally, the retention time of threonine in the sample hydrolysate was consistent with that of threonine generated through acid hydrolysis of a PT standard (Fig. 1C). We also confirmed that hydrolysis was complete through analyzing a PT standard both before and after treatment (supplemental Fig. S5).

Fatty acyl (FA) compositions of PT in human blood were determined using MS^*n*^ scans. Initially, these were collected as MS^2^ for each PT precursor ion. However, due to some coelution of PT with PS, these spectra also contained ions originating from odd-chain FA-containing PS species, as indicated by an ion resulting from a neutral loss of 87 Da (Fig. 1D, E). Thus, MS^3^ was instead used to characterize the FA composition of PT species, from either whole blood or washed platelet lipid extracts. Here, product ions resulting from threonine head group loss were selected as second precursors for further fragmentation (Fig. 1F, G, supplemental Figs. S6 and S7). For all, apart from PT 40:3 which was extremely low abundance, the MS^3^ evidences both FA chains and the neutral loss of the carboxylates and ketenes for the *sn*-1 FA. As we were unable to obtain a convincing PT 40:3 MS^3^ spectrum, we propose this low abundance PT is putatively identified based on retention time comparison with other PT species. Further confirmation of structural assignments was provided by spiking a platelet lipid extract with two PT standards, PT 18:0/18:1, and PT 16:0/18:1 and showing co-elution (supplemental Fig. S8). High resolution MS/MS for these standards are also provided (supplemental Fig. S9). Overall, PS and PT chain composition was similar and largely typical of mammalian phospholipids, in that even-chain FA predominated. Some precursor ions were comprised of more than one molecular species (Table 1). This was more common for PS compared to PT, likely due to the former being in higher abundance.

**Table 1:** Fatty acid compositions of PS and PT species in human platelets and leukocytes

Lipid	FA composition (platelets)	FA composition (leukocytes)
PS 34:1	16:0_18:1 & 16:1_18:0	16:0_18:1 & 16:1_18:0
PS 36:1	18:0_18:1	18:0_18:1
PS 36:2	18:1_18:1 & 18:0_18:2	18:1_18:1 & 18:0_18:2
PS 38:3	18:0_20:3	18:0_20:3
PS 38:4	18:0_20:4	18:0_20:4
PS 38:5	18:1_20:4	18:1_20:4 & 18:0_20:5 & 16:0_22:5
PS 40:3	18:0_22:3 & 18:1_22:2 & 20:0_20:3	18:0_22:3 & 18:1_22:2 & 20:0_20:3
PS 40:4	18:0_22:4 & 18:1_22:3 & 20:0_20:4	18:0_22:4 & 18:1_22:3 & 20:0_20:4
PS 40:5	18:0_22:5 & 18:1_22:4 & 20:1_20:4	18:0_22:5 & 18:1_22:4 & 20:1_20:4
PS 40:6	18:0_22:6 & 18:1_22:5 & 20:2_20:4	18:0_22:6 & 18:1_22:5 & 20:3_20:3
PT 34:1	16:0_18:1	16:0_18:1
PT 36:1	18:0_18:1	18:0_18:1
PT 36:2	18:1_18:1 & 18:0_18:2	18:1_18:1
PT 38:3	18:0_20:3	18:0_20:3
PT 38:4	18:0_20:4	18:0_20:4
PT 38:5	18:1_20:4	N/A (low signal)
PT 40:3	N/A (low signal)	N/A (low signal)
PT 40:4	18:0_22:4 & 20:0_20:4	N/A (low signal)
PT 40:5	18:0_22:5 & 20:1_20:4	18:0_22:5
PT 40:6	18:0_22:6	N/A (low signal)

FA composition was determined using LC-MS/MS on the 6500 QTRAP system as described in Supplementary Methods. Representative chromatograms and MS3 spectra of PT species are available in supplemental Figs. S6 and S7.

Next, PT and PS were quantified in whole blood from three healthy donors using HILIC-LC-MS/MS (Fig. 1H). PT 38:4 (18:0_20:4) was the most abundant, followed by PT 36:1 (18:0_18:1), PT 40:6 (18:0_22:6), PT 40:5 (18:0_22:5), and PT 40:4 (18:0_22:4). This profile will be most representative of erythrocytes with minor contributions from platelets and leukocytes, based on their respective counts in blood. The highest abundant species was PS 38:4 (18:0_20:4), followed by PS 40:6 (18:0_22:6), PS 40:5 (18:0_22:5), PS 36:1 (18:0_18:1), and PS 40:4 (18:0_22:4), consistent with a red blood cell PS profile in a previous report (31). Notably, PT and PS have similar FA compositions, but PS contains a higher proportion of 22-carbon FA-containing species (as a percentage of total) than PT, and its concentration in whole blood was approximately 100-fold higher than PT (Fig. 1H).

### PT profiles of human platelets, leukocytes, and extracellular vesicles

**Fig. 2:**
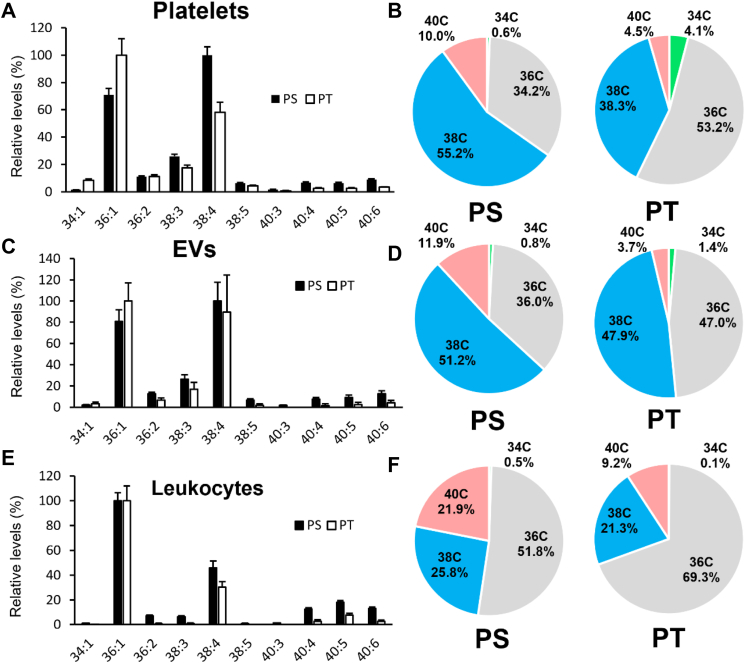
Comparison of PT and PS molecular species in human platelets, EV, and leukocytes. Lipid extracts of platelets, leukocytes, and EVs from healthy volunteers from the clinical cohort were analyzed for PT and PS using HILIC-LC-MS/MS as described in Supplementary Methods and normalized to the A/IS values for the most abundant species detected for either PT or PS. For bar charts (A, C, E), data are represented as mean ± SEM (n = 24). 100% is set as the level of the most abundant PT or PS in the relevant sample type. For pie charts (B, D, F), A/IS values of molecular species with the same FA carbon number were summed and used to calculate levels relative to the total. EV, extracellular vesicle; LC-MS/MS, liquid chromatography–tandem mass spectrometry; PS, phosphatidylserine; PT, phosphatidylthreonine.

Next, the molecular compositions of PT and PS in platelets, leukocytes, and EV were compared using LC-MS/MS (Fig. 2). We focused on leukocytes and platelets, since they are considered to provide the pro-coagulant lipid surface, with EVs have been proposed to also play a role in thrombosis in CAD (32). Platelet PS was consistent with Leidl *et al.* (31), where PS 38:4 was the most abundant followed by PS 36:1 (Fig. 2A). In contrast, the most abundant PT was PT 36:1 followed by PT 38:4 (Fig. 2A, B). EVs showed a similar pattern to platelets (Fig. 2C, D), consistent with most EVs in the healthy circulation being platelet-derived (33). For leukocytes, the 36:1 species were the most abundant for both PS and PT (Fig. 2E, F). In all three sample types, pie charts show that PT was more enriched in shorter 34 and 36C (total carbon length) FA than PS, while PS was enriched in longer 38C and 40C species (Fig. 2B, D, F). For isolated cell samples, we also wished to determine relative abundance of PS to PT, similar to the analysis for blood lipids shown in Fig. 1H. However, PS[D7] internal standard had been added to pre-extracted cohort samples, and so only A/IS values were generated for the cohort. This was because these samples were from an existing cohort for which lipid extracts had been already generated for analysis of oxidized phospholipids, and PS[D7] had not been added for that purpose. To address this, we first compared PT and PS for detection using serial dilutions of the same molecular species (18:0/18:1) and found that they were detected with a relatively similar sensitivity on our MS (supplemental Fig. S10). This allowed a rough visual comparison of A/IS values for PT and PS in platelets, leukocytes and EVs (supplemental Fig. S11). Similar to whole blood, abundance of PT is seen to be around 100-fold lower than PS in all these tissues.

### Platelets metabolize and externalize PT following thrombin activation

**Fig. 3:**
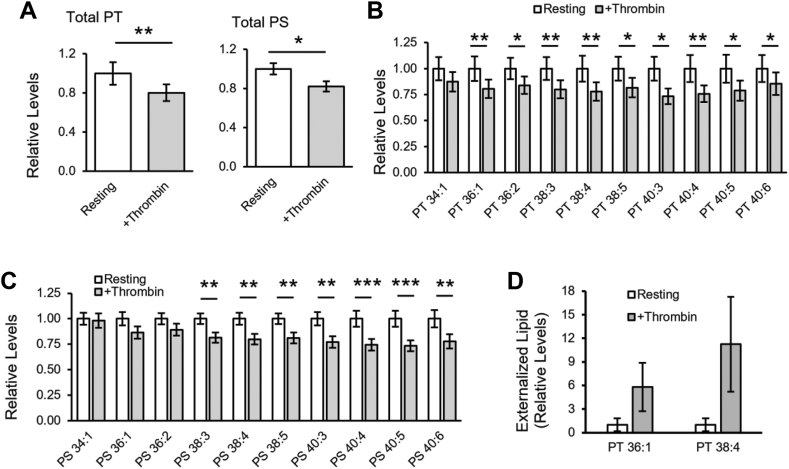
Thrombin stimulates metabolism of PT/PS in platelets, and PT is externalized on thrombin activation. A: Thrombin activation stimulates metabolism of PT and PS. Relative levels of total PT and PS molecular species were determined in resting and thrombin-activated platelets (n = 24 healthy donors) using LC-MS/MS as described in Supplementary Methods. Levels are expressed relative to PT or PS in resting cells. B, C: Individual PT and PS molecular species are metabolized following thrombin activation of platelets. Data were generated as in (A) above but shown here for individual molecular species (n = 24). D: PT is externalized following thrombin activation of platelets. Resting or thrombin-activated platelets were treated with sulfo-NHS-biotin to derivatize PT in the outer membrane leaflet, then lipids were extracted and analyzed using RP-LC-MS/MS as described in Supplementary Methods (n = 3, mean ± SEM). Levels are expressed relative to biotinylated PT in resting platelets. Statistical significance was determined using paired t-tests (∗*P* < 0.05, ∗∗*P* < 0.01, ∗∗∗*P* < 0.001). LC-MS/MS, liquid chromatography–tandem mass spectrometry; PS, phosphatidylserine; PT, phosphatidylthreonine.

Next, the effect of thrombin activation on platelet PT was tested (Fig. 3). First, PT and PS levels were compared in resting and thrombin-activated platelets from the healthy volunteer cohort group using HILIC-LC-MS/MS. Total levels decreased in platelets following thrombin activation (−19.8% and −17.8%, for PT and PS, respectively), with several individual species being significantly different (Fig. 3A–C). This suggests that PT and PS are both metabolized on activation. Next, externalized PT was determined in platelets from three healthy volunteers using sulfo-NHS-biotin derivatization followed by LC-MS/MS analysis, as described previously for PE and PS (34). Two biotinylated PT species were detected, 36:1, 38:4, and these were increased following thrombin activation (Fig. 3D, supplemental Table S3, supplemental Figs. S12 and S13). This demonstrates that PT, like PS, is externalized in activated platelets, suggesting it may also play a role in coagulation reactions.

### PT promotes coagulation in vitro through enhancement of FXa:FVa but not TF:FVIIa

**Fig. 4:**
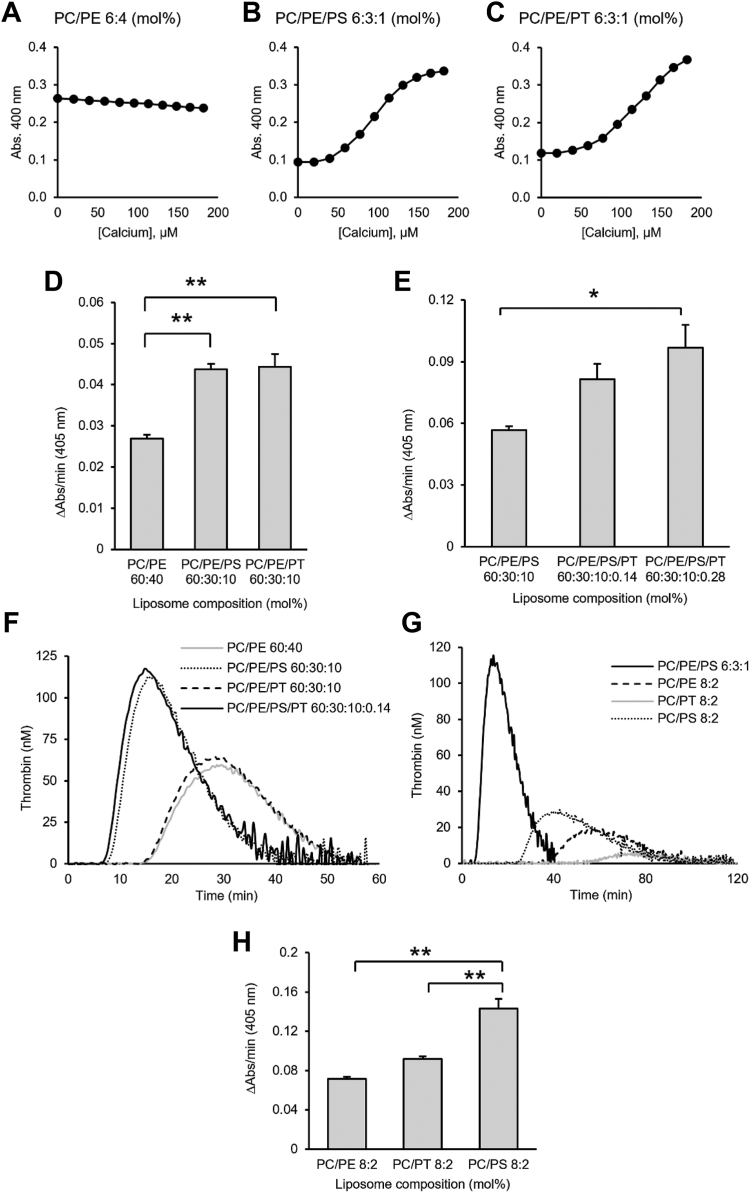
PT liposomes bind calcium and support prothrombinase activity in vitro. Liposomes of varying compositions were prepared by extrusion and tested for Ca^2+^ binding and coagulation, as described in Supplementary Methods. A–C: Turbidimetric Ca^2+^-binding assay shows binding to PT- and PS-containing liposomes. Liposomes (1 mM) were titrated with CaCl_2_ (2 mM), and change in absorbance at 400 nm was monitored following each addition of calcium. Data points in binding curves represent the average of two technical replicates. D, E: PT liposomes enhance prothrombinase activity in vitro. Prothrombinase assay: Liposomes (25 μM) were incubated with FII (200 nM), FVa (3.0 nM), FXa (10 nM), and 1 mM CaCl_2_ for 5 min. The reaction was stopped and FIIa generation was monitored for 50 min as described in Supplementary Methods (mean ± SEM, n = 3). Statistical significance was determined using one-way ANOVA and post-hoc Tukey test (∗*P* < 0.05, ∗∗*P* < 0.01). F, G: Liposomes containing PT do not enhance TF-dependent coagulation in platelet-poor plasma. Liposomes (10 μM) containing embedded TF (50 pM) were tested for their ability to support coagulation using calibrated automated thrombinography (CAT) as described in Supplementary Methods. Thrombin generation was calculated automatically using a calibration reference curve. Trace represents the average of three independent measurements. H: PT does not enhance coagulation in the extrinsic tenase assay. Liposomes (25 μM) with embedded TF (1 nM) were tested for their ability to stimulate coagulation using an in vitro extrinsic tenase assay as outlined in Supplementary Methods (mean ± SEM, n = 3). Statistical significance was determined using one-way ANOVA and post-hoc Tukey tests (∗*P* < 0.05, ∗∗*P* < 0.01). Where no significance is shown, the differences between groups were not significant. PS, phosphatidylserine; PT, phosphatidylthreonine; TF, tissue factor.

PS externalization is required on the surface of platelets to sustain coagulation, and PE synergizes with PS to enhance coagulation factor binding (35, 36). As PT was also externalized (Fig. 3D), we next explored its ability to bind coagulation factors and support their activities. Here, liposomes containing combinations of PC, PE, PS and PT were first tested for Ca^2+^ binding, since Ca^2+^ bridges mediate Gla domain PS interactions (37). Liposomes were titrated with Ca^2+^ and monitored for an increase in absorbance at 400 nm. Liposomes containing PC and PE alone did not bind Ca^2+^ (Fig. 4A). In contrast, liposomes containing PC and PE, supplemented with PS showed dose-dependent changes in absorbance, as expected based on the known ability of PS to bind Ca^2+^ (Fig. 4B) (38, 39). In liposomes where the PS was replaced with PT, a similar Ca^2+^ binding curve was observed (Fig. 4C). These data indicate that Ca^2+^ is bound by liposomes containing anionic PT, similar to PS, but not the neutrally-charged PE. Further studies will be undertaken to determine affinity and co-operativity between PS and PT for calcium binding.

Next, liposomes were tested for coagulation factor activation using a purified protein prothrombinase (FXa:FVa) assay, CAT, and an extrinsic tenase (TF:FVIIa) assay (40). First, we found that PC/PE/PT liposomes, supported FXa:FVa activity to the same extent as PC/PE/PS liposomes and significantly better than PC/PE liposomes (Fig. 4D). This indicates that PT can act similar to PS, and even replace it in supporting prothrombinase activity. Next, to test whether physiological amounts of PT could support coagulation in a membrane that approximates the composition of a platelet, PT was included at low amounts (0.14% and 0.28%) in liposomes containing physiological amounts of PS (10%). Here, addition of small amounts of PT to PC/PE/PS liposomes enhanced their ability to support FXa:FVa activity (Fig. 4E). Next, the ability of PT to support coagulation was tested using the CAT assay with platelet-poor plasma (PPP), which contains all required factors. Here, coagulation is triggered by tissue factor incorporated into the liposomes. In contrast to our findings with prothrombinase alone, in plasma we found that PC/PE/PT liposomes poorly stimulated thrombin generation, and addition of PT to PC/PE/PS liposomes did not augment thrombin generation (Fig. 4F). To test this further, we simplified the system, by removing PE, and found that PC/PT liposomes bearing TF poorly generated thrombin in PPP compared to PC/PE/PS, PC/PS, and PC/PE liposomes (Fig. 4G). To follow on from this, being consistent with the previous experiment, to allow us to specifically look at PT, we tested extrinsic tenase (TF:FVIIa) in a purified system. Here, PC/PT liposomes containing TF poorly supported extrinsic tenase (TF:FVIIa) activity, unlike PC/PS (Fig. 4H). Last, we returned to the prothrombinase assay, this time maintaining the lipid composition used for extrinsic tenase (no PE), so that the impact of PT alone could be tested. Here, we found that PT dose dependently enhances prothrombinase activity, even in the absence of PE (supplemental Fig. S14). Overall, these data suggest that PT promotes coagulation through enhancement of FXa:FVa activity but not TF:FVIIa activity.

### PT strongly binds FVa but poorly binds FX and prothrombin (FII)

**Fig. 5:**
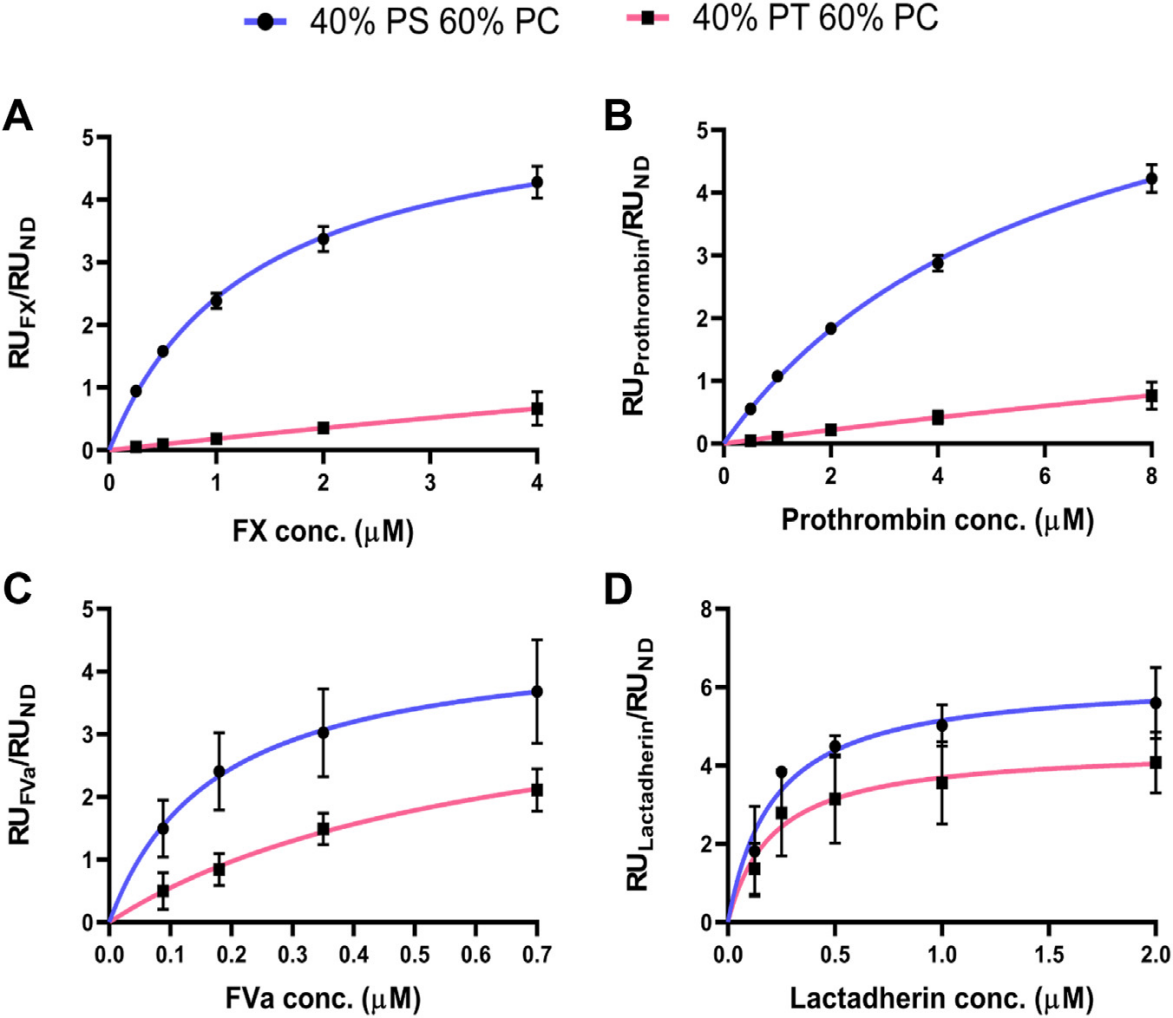
Binding isotherms of proteins with nanodiscs containing phosphatidylthreonine or phosphatidylserine. A, B: Gla domain-containing proteins: FX (A) and prothrombin (B) do not achieve saturable binding to PT nanodiscs, unlike PS nanodiscs. C, D: C2-like domain-containing proteins: FVa (C) and lactadherin (D) achieve saturable binding to both PT nanodiscs and PS nanodiscs. Nanodiscs were prepared and tested for their ability to bind coagulation proteins and lactadherin using SPR as described in Supplementary Methods. Nanodisc compositions denote lipid mol% values: 40% PS 60% PC (blue) and 40% PT 60% PC (pink). Data represent mean ± SEM (n = 3, technical replicates). Binding affinities are available in Table 2. FX, factor X; FVa, activated factor V; PC, phosphatidylcholine; PS, phosphatidylserine; PT, phosphatidylthreonine.

To determine why PT supports FXa:FVa activity but not TF:FVIIa activity, we investigated the binding of FX, prothrombin and FVa to nanodiscs containing PC:PT (60:40) using surface plasmon resonance. Nanodiscs containing PC:PS (60:40) were used to compare the binding propensity of the clotting proteins toward PS versus PT (Fig. 5, exemplary sensorgrams in supplemental Fig. S15). We note that experimental conditions were adjusted to take into account the logistics of working with the nanodisc system. First, when we examine binding of clotting proteins to nanodiscs, a higher relative composition of anionic lipids than would be used in liposomes is needed (41). We suspect this to be because anionic lipids like PS can cluster into PS-rich nanodomains in the presence of both calcium ions and PS-binding proteins. Therefore, the nanodisc bilayer is mimicking a single clustered, PS-rich nanodomain and therefore requires a higher PS concentration than a liposome (41). We took the same approach with PT in our studies in this paper. Second, since the nanodiscs have a diameter of 10 nm and contain only ∼60–70 phospholipid molecules per leaflet, it is not possible to test 0.14% (mol% not mol fraction) or 0.28% PT compositions. The liposomes used in for the prothrombinase assay are around 100 nm, making the latter a more suitable assay to test effects of tiny amounts of lipids. Note also that in all experiments, controls containing 100% PC were used to calculate background which was subtracted from experimental data.

Using nanodiscs, FX and FII did not achieve a saturable steady state binding with nanodiscs containing PC:PT as compared to PC:PS (Fig. 5A, B and Table 2). However, for FVa, saturable binding to PC:PT with a binding affinity of 0.520 ± 0.310 μM was observed (Fig. 5C and Table 2). That said, we note some binding of FVa to the empty flow cell and 100% PC nanodiscs (supplemental Fig. S15C), and this can be further explored in future work. In the context of the coagulation assays shown above, this suggests that PT supports FXa:FVa activity through the recruitment of FVa, and the inability of PT to support TF:FVIIa activity could be due to the poor binding of FVIIa’s Gla domain to PT, but this requires direct evidence with experiments on FVIIa.

**Table 2:** Binding affinities of clotting proteins in the prothrombinase complex and lactadherin to lipid nanodiscs

Protein	K_d_ (μM)		
	40% PS 60% PC	40% PT 60% PC	20% PT 20% PS 60% PC
Factor X	1.33 ± 0.191	N/A^a^	2.04 ± 1.07
Prothrombin	6.23 ± 0.921	N/A^a^	8.66 ± 0.565
Factor Va	0.174 ± 0.130	0.520 ± 0.310	0.645 ± 0.501
Lactadherin	0.212 ± 0.088	0.208 ± 0.153	0.280 ± 0.0944^b^

Data are mean ± standard error of three technical replicates. Binding affinities were determined as described in Supplementary Methods. Binding isotherms are in Fig. 5 and exemplary sensorgrams in supplemental Fig. S15.

a No saturable binding observed.

b Binding affinity measured only in one experiment.

### PT binds C2-like domains while Gla domains are selective for PS

Since both FX and FII bind membranes via Gla domains whereas FVa binds via the C2-like domain, we questioned whether the C2-like domains are less selective for PS as compared to the Gla domains. To address this, binding studies were conducted with lactadherin since this protein also binds to PS via the C2-like domain (42). Here lactadherin’s C2-like domain bound to PC:PS (60:40) and PC:PT (60:40) with binding affinities of 0.212 ± 0.088 μM and 0.208 ± 0.153 μM, respectively (Fig. 5D and Table 2). These data indicate that C2-like domains bind PT and PS in PC containing membranes, with comparable high affinities, while Gla domains only bind PS.

### FX and prothrombin bind membranes containing both PS and PT

Previous studies showed that FX’s Gla domain requires a minimum of one PS molecule in membranes containing excess PE (43). Since we did not observe saturable binding for FX and prothrombin (FII) with PC:PT (60:40) and based on the observation that PT when added to PS liposomes enhances prothrombinase activity (Figs. 4E and 5A, B), we hypothesized that membranes comprising both PS and PT would support FX and prothrombin binding. To test this, we conducted further binding studies of FX, prothrombin and FVa with nanodiscs containing PC:PS:PT (60:20:20). These show that two Gla-domain containing FX and FII proteins bound to the nanodiscs with affinities of 2.04 ± 1.07 μM and 8.66 ± 0.565 μM, respectively (Table 2). However, whether PT synergizes with PS to enhance the binding of Gla domain containing clotting proteins to anionic membrane surfaces remains unclear and requires further investigation.

### PT is significantly elevated in platelets and EVs from patients with coronary artery disease

**Fig. 6:**
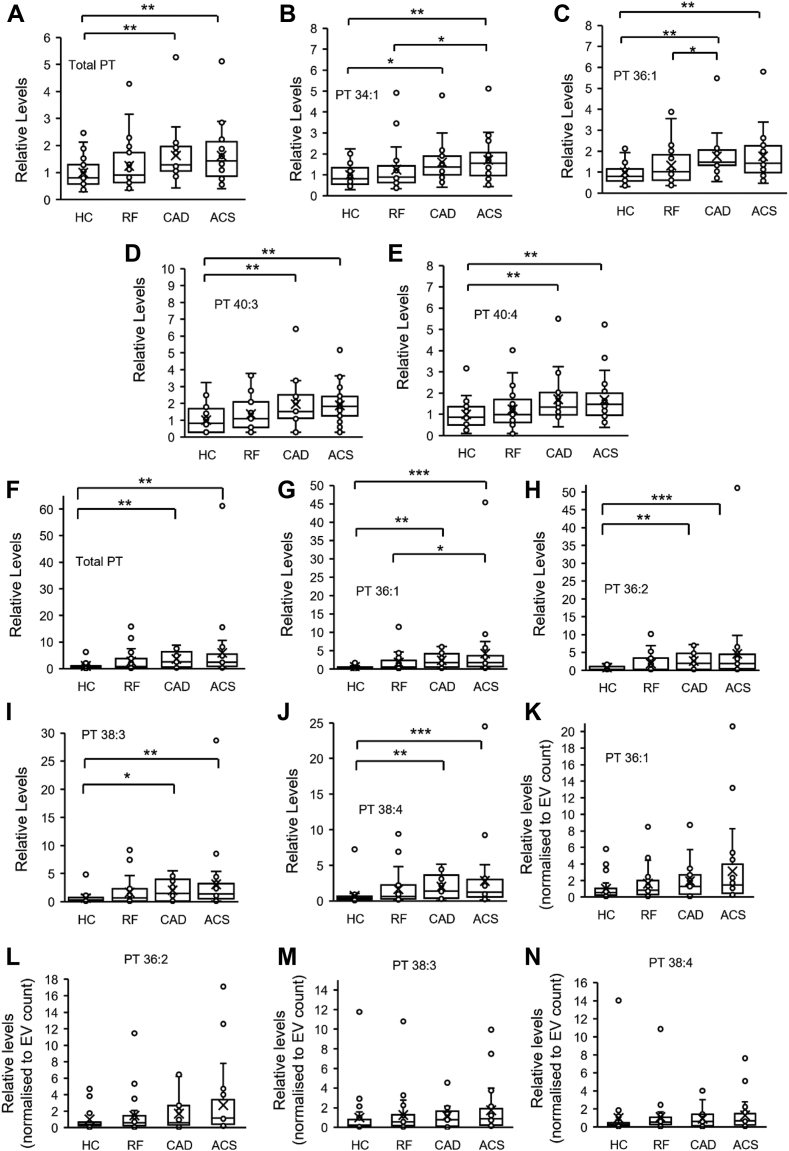
PT is significantly elevated in platelets and EV from patients with arterial vascular disease. Lipids were analyzed for PT using HILIC-LC-MS/MS, in lipid extracts from platelets or EV from a clinical cohort as described in Supplementary Methods. Analyte peak areas were integrated and ratios of analytes to internal standards (A/IS) calculated. Total PT levels were calculated by summing A/IS values of individual species for the respective samples. A–E: Platelet PT is significantly elevated in platelets from patients with arterial vascular disease. Values for either total PT or individual molecular species are normalized to the mean of the HC group. F–J: EV PT is significantly elevated in patients with arterial vascular disease. Values for either total PT or individual molecular species are normalized to the mean of the HC group. K–N: Higher levels of PT in EV is related to the higher EV count in plasma of patients with vascular disease. PT in EV was normalized using EV counts obtained using nanoparticle tracking analysis. Values were normalized to the mean of the HC group. Statistical significance was determined using Kruskal-Wallis H test (∗*P* < 0.05, ∗∗*P* < 0.01, ∗∗∗*P* < 0.001). Where no significance is shown, the differences between groups were not significant. HC, healthy controls (n = 24); RF, risk factors with no significant coronary artery disease (n = 23); CAD, coronary artery disease but no acute coronary syndrome (n = 19); ACS, acute coronary syndrome (n = 24). Data are presented as box and whisker plots, in which the box edges indicate the interquartile range (IQR) with the median line inside the box. Whiskers indicate 1.5 times the IQR, and “X” represents the mean. EV, extracellular vesicle; LC-MS/MS, liquid chromatography–tandem mass spectrometry; PT, phosphatidylthreonine.

Since PT can support coagulation, it may be altered in diseases where patients are at higher thrombotic risk. To test this, PT was measured using LC-MS/MS, in platelets and EVs isolated from a clinical cohort of coronary artery disease patients. In platelets, following activation by thrombin, PT was partially metabolized, most likely by phospholipases. However, the magnitude of this was similar for all patient groups (supplemental Fig. S16). In contrast, total platelet PT was significantly higher in coronary artery disease (CAD) and acute coronary syndrome (ACS) patient groups than healthy volunteers (HC) (Fig. 6A). This was further reflected in significantly higher levels of individual PT species: PT 34:1, 36:1, 40:3, and 40:4, as well as non-significant trends for other PTs (Fig. 6B–E and supplemental Fig. S17). PT was also significantly higher in EVs (isolated from plasma) from CAD and ACS patient groups compared to healthy volunteers (Fig. 6F–J). Due to the lower levels of lipid in these samples, only four PT molecular species (PT 36:1, 36:2, 38:3, and 38:4) were detected in >50% of EV samples, and all were significantly elevated in disease groups. EV numbers are higher in patients with cardiovascular disease, including in this cohort (44). To investigate whether this accounted for elevated PT levels, lipid levels were normalized to EV counts, previously published in a related study using this cohort (29). After normalization, PT species were no longer significantly different in the disease groups (Fig. 6K–N), indicating that higher levels of PT relate directly to higher EV counts in thrombotic disease. For both platelets and EVs, a small number of outliers were noted for PT and PS. We have repeated our statistical analysis after removal of outliers, and for platelets but this had no impact on significance (data not shown), and so data including outliers is shown. For EVs, removing outliers led to no change for significance for values that were not normalized to EV counts (not shown). However, when outliers were removed from data where values had been normalized to EV counts, CAD and ACS groups became significantly elevated in comparison to HC (supplemental Fig. S18). This suggests that EV from patients with CVD may contain slightly elevated PT, however since this was only apparent after removal of outliers, further studies are required to determine if it is biologically relevant. Last, for PT levels, we found no significant differences between male or female participants in the HC, RF or ACS groups, while there were too few females in the CAD group to test (supplemental Fig. S19).

## Discussion

Here, we describe PT as an endogenous aPL in human blood cells, including platelets, leukocytes, and EV, and show that it supports coagulation factor activities in vitro as well as being elevated in human thrombotic disease. Our data reveal PT as a novel pro-coagulant phospholipid, supporting factor binding to membranes, alongside the canonical activities of PS and PE (36). As EV can originate from many vascular cell sources, their PT could come from a variety of cell types.

The well-known binding of coagulation factors to PS is mediated by either γ-carboxyglutamate-rich (Gla) domains, found in FII, FVII, FIX, and FX, or discoidin (C2-like) domains in FVa and FVIIIa (4, 5, 6). Here, PT was found to strongly bind FVa and lactadherin (Fig. 5C, D and Table 2), both of which contain C2-like domains. By contrast, PT poorly bound the Gla domain-containing FX and FII. Consequently, PT enhanced the activity of the prothrombinase complex but not the extrinsic tenase complex (Fig. 3D, H). Thus, PT was procoagulant through enhancement of prothrombinase, mediated through interactions with FVa. The CAT assay (using PPP) is very different to the purified prothrombinase assay, being dependent on tissue factor/FVIIa for activation of FX in plasma and involving multiple coagulation complexes. There are several reasons why we may see differences between the CAT assay and assays using purified clotting factors. First, in this assay, high levels of Xa are generated by the tissue factor/FVIIa complex as a first step, so the rate of formation of Xa drives the generation of thrombin. In Fig. 4F, we show that this complex is sensitive to PS but not PT, similar to the CAT data. This is likely due to the fact that PT appears to not bind Gla domains (VIIa), instead binding C2-like domains (FVa). Thus, the CAT data is consistent with the purified enzyme assay showing that tissue factor/FVIIa complex is sensitive to PS but not PT (Fig. 4H). Second, the CAT assay is influenced by negative feedback mechanisms such as TFPI, protein S and protein C. TFPI has negatively charged domains (Kunitz), while proteins S and C contain Gla domains. Thus, it is possible that PT can enhance activity of pathways that limit thrombin generation in this assay, differently to PS. These ideas will be tested in future studies beyond the scope of this manuscript. Last, it is possible that PT also binds FVIIIa since it also contains a C2-like domain (45), but this also remains to be tested.

Physiological amounts of PT, as judged by the relative levels of PT 36:1 compared to PS 36:1 in platelets (supplemental Fig. S11), directly enhanced the ability of PS to support prothrombinase (Fig. 4E). Thus, despite its low abundance compared to PS in vivo, PT can support coagulation reactions in tandem with PS. The mechanism by which PT enhances the activity of the prothrombinase complex (when present alongside PS in liposomes) is not known. Other phospholipids including PE synergize with PS to enhance FX activation by extrinsic tenase (46). Thus, while PT alone can support prothrombinase activity through binding FVa, in combination with PS it may also enhance binding of Gla domains (of FXa and FII) to PS membranes (Fig. 4D, E), but this requires further investigation. Overall, our data shows that the binding of C2-like domains occurs on surfaces containing either PT or PS with similar high affinities, and this is distinct from the binding behavior of Gla domains to PS. The lack of interaction of PT with Gla domains demonstrates how addition of a methyl group to the β carbon of the PS serine head group hinders binding and highlights the specificity of FX and FII Gla domains for PS. Importantly, this data showed that the increased prothrombinase activity seen is likely mediated via C2-like domains, independent of prothrombin binding.

Although we did not include data on PE in this study, it was included in our biochemical assays, to approximate a physiological membrane composition. PE is present at levels that are higher than PS in white cells and platelets (31). In that study, LC-MS/MS was used to directly compare the levels of all PL classes in platelets, erythrocytes, and individual leukocyte populations. Notably, PE accounted for around 15%–30% of the total PL pool in comparison to PS, which accounted for around only 9%–10%. We also previously characterized molecular species of PE, and found similar profiles for both PS and PE in platelets (47). Notably, PE is also present in plasmalogen forms, which are often more abundant than diacyl species.

Coronary artery disease (CAD) and acute coronary syndrome (ACS) patients are at increased risk of thrombosis, and previous studies have demonstrated increased EV levels, as well as platelet hyperactivity and lipid dysregulation in this patient group (48, 49, 50, 51). Here, we show that PT is significantly higher in platelets from these patients compared to healthy volunteers (Fig. 6A–E). The reason behind these increased levels is unknown but suggests biochemical changes in platelets in CAD/ACS. A higher proportion of immature (reticulated) platelets may contribute, as they are characterized by a larger size, higher amounts of membranous organelles (ER, mitochondria, granules) compared to mature platelets, and have been shown to be elevated in CAD and ACS (52, 53, 54, 55, 56). We found that higher EV counts in plasma of patients led to higher levels of EV-derived PT. Thus, overall patients with CAD have higher levels of PT originating from both platelets and EV in their circulation and given the pro-coagulant activity of this lipid, PT may contribute to elevated thrombotic risk in this patient group.

The biochemical origin of PT in mammalian cells is not yet known. PS is synthesized in human cells by two phosphatidylserine synthase (PSS) enzymes, PSS1 and PSS2, through base-exchange reactions. PSS1 converts PC and PE into PS in vitro, but prefers PC in vivo, while PSS2 converts PE into PS and exhibits a preference for PE with docosahexaenoic acid (FA 22:6) at the *sn*-2 position (57, 58). While PSS1 is encoded by a housekeeping gene and is thus present in virtually all cells (26), little is known about the distribution of PSS2 in circulating blood cells. PT has been proposed to be synthesized by PSS synthases through a base-exchange reaction (19), but it is unknown which isoform is relevant in vivo. Our results demonstrate that PT profiles of platelets and leukocytes are more enriched in 34-carbon and 36-carbon and less enriched in 38-carbon and 40-carbon species compared to PS (Fig. 2B, F). This, along with the observation that PC species are more enriched in shorter chain species compared to PE species in circulating blood cells (31, 59), suggests that PT may be synthesized predominantly by PSS1 from the PC pool, whereas PS could be synthesized by both PSS isoforms from PC and PE pools.

The platelet lipidome undergoes significant changes following thrombin activation. These include the release of polyunsaturated fatty acids (PUFA, particularly FA 20:4) from phospholipid (PL) pools, oxygenation of PUFA into oxylipins, and esterification of oxylipins back into PL to form enzymatically oxidized PL (60, 61, 62). Platelet PC, PE, and phosphatidylinositol (PI) containing FA 20:4 all decrease following thrombin activation (60, 62). Additionally, activated platelets mobilize PS and PE from the inner plasma membrane leaflet to the outer leaflet (47). However, changes in PS levels are less studied. A previous study showed that thrombin activated platelets incorporated external labelled glycerol into PS, but this was transient with maximal rates in the first 2 min (63). Furthermore, this experiment did not directly measure endogenous PS biosynthesis because exogenous glycerol was used. In contrast, other studies reported no changes in total platelet PS after 1–15 min activations with thrombin (59, 60, 62). Here we show that platelet levels of several PS species decreased following a 30 min incubation with thrombin, suggesting that hydrolysis may occur later during activation (Fig. 3A, C). Similarly, PT also decreased following thrombin activation (Fig. 3A, B), and like PS, it was externalized by activated platelets (Fig. 3D). PT may be maintained in the inner membrane and externalized into the outer leaflet through the same mechanisms as PS and PE, i.e., through aminophospholipid flippase and scramblase, but this requires further investigation.

In conclusion, PT molecular species were characterized in human blood, platelets, leukocytes and EVs, and PT metabolized and externalized by platelets following thrombin activation. It enhances prothrombinase activity in vitro through the binding of FVa, and its levels are significantly higher in platelets and EVs from CAD patients. Future studies will address the metabolism of PT in activated blood cells (e.g., the generation of lysoPT), in vivo roles in coagulation and apoptosis owning to its ability to bind C2-like domains found in clotting factors and other proteins, and its link to vascular disease.

## Data availability

All data produced in the present study are available upon reasonable request to the authors (contact Ali A. Hajeyah, ali.hajeyah@ku.edu.kw).

## Supplemental data

This article contains supplemental data (28, 29, 30, 34, 38, 39, 43, 47, 64, 65, 66, 67, 68, 69, 70, 71, 72).

## Conflict of interest

The authors declare that the research was conducted in the absence of any commercial or financial relationships that could be construed as a potential conflict of interest.
