# Supplementary material for: Phosphatidylthreonine is a procoagulant lipid detected in human blood and elevated in coronary artery disease
Source: J Lipid Res. Author manuscript; Available in PMC 2024 Feb 5. (PMC10809103; doi:10.1016/j.jlr.2023.100484)
Supplement: Supplementary [file EMS193720-supplement-Supplementary.docx]

**Supplementary Methods, Figures, Tables**

**Supplementary Materials and Methods**

*Blood collection from healthy volunteers*. Blood was drawn by venipuncture using 21-gauge butterfly needles into syringes containing 3.2 % trisodium citrate (9:1 v/v) and 50 µg/mL corn trypsin inhibitor (CTI, Haematologic Technologies (Vermont, USA). 200 µL aliquots were snap frozen in liquid nitrogen and stored at -80 °C until use. To generate platelet-poor plasma (PPP), blood volunteers was drawn by venipuncture using 21-gauge butterfly needles into syringes containing trisodium citrate (final: 0.32 % w/v) and CTI (final: 20 µg/mL) then transferred into falcon tubes. PPP was prepared from whole blood using two centrifugation steps (1730 *g*, 10 min, 21 °C). PPP from 25 donors was pooled, aliquoted and frozen at -80 °C until use in calibrated automated thrombinography (CAT). The human studies conducted and reported herein abide by the Declaration of Helsinki.

*Platelet isolation, thrombin activation, and NHS-biotin derivatization.* Washed platelets from healthy volunteers were prepared from whole blood drawn into syringes containing acidified citrate dextrose (ACD, 85 mM trisodium citrate, 65 mM citric acid, and 100 mM glucose (pH 5.4)) at a ratio of 8.1 parts whole blood to 1.9 parts ACD, as described previously (1), and resuspended in Tyrode’s buffer (134 mM NaCl, 12 mM NaHCO_3_, 2.9 mM KCl, 0.34 mM Na_2_HPO_4_, 1 mM) at 2 x 10^8^/mL. One set of 1 mL aliquots of platelets was left on the bench at 21 °C (resting platelets). Another set was recalcified with 1 mM CaCl_2_, incubated at 37 °C for 5 min, then activated with bovine thrombin (0.2 U/mL) at 37 °C for 30 min (activated platelets). Resting and activated platelets (4 x 10^7^) were treated with sulfo-NHS-biotin to derivatize external (plasma membrane) leaflet lipids or NHS-biotin to derivatize all lipids containing primary amine groups, as previously described (2). Following derivatization, phosphate-buffered saline (PBS, pH 7.4, Thermo Fisher Scientific) was added to samples to make 1 mL for lipid extraction, with samples stored at -80 °C until analysis.

*Clinical cohort.* Participants were recruited from Cardiff University and Cardiff and Vale University Health Boards, as described (3). Ethical approval was from Health and Care Research Wales (HCRW, IRAS 243701; REC reference 18/YH/0502). Study groups of at least 20 were aimed for based on a previous study in venous thrombosis (4). Age and gender-matched individuals were recruited into one of the following four groups: *(i) Acute Coronary Syndrome (ACS)*: Participants were identified on in-patient cardiology wards using diagnostic tests (ischemic ECG changes, raised troponin level above normal laboratory defined range) and clinical assessment by the cardiology team. All were recruited within 48 hours of the index event prior to any revascularization/angioplasty. *(ii)* *Significant coronary artery disease (CAD):* Patients attending an elective coronary angiogram to assess for symptoms of stable angina in the absence of a history of acute coronary syndrome were recruited. Coronary angiography demonstrated lesions requiring revascularization on anatomical/physiological criteria as defined by guidelines from the European society for cardiology (ESC, 2018) (5). *(iii)* *Risk-factor controls with no significant CAD (RF):* This group includes patients attending for a diagnostic coronary angiogram with any risk factors for ischemic heart disease (a clinical diagnosis of hypertension requiring therapy, diabetes types 1 or 2, hypercholesterolemia [total cholesterol > 6 mM], smoking, chronic kidney disease stage 3 or more, or combination thereof) but whose coronary angiogram demonstrates no significant coronary artery disease, defined as not requiring revascularization on anatomical/physiological criteria as per the ESC 2018 guidelines (5). *(iv)* *Healthy controls (HC):* Participants had no history of ischemic heart disease or its risk factors, were never-smokers, and were not on anti-platelet agents, anti-coagulants, anti-hypertensives, or statins. They were identified from the workplace or were volunteers from partner studies such as ‘HealthWise Wales’ (6). Inclusion criteria were aged 18 yrs. or older, acute coronary syndrome in ACS group, and no history of ACS in the others. Exclusion criteria were: diagnosis of infective endocarditis or atrial fibrillation, or inability to consent to study. Here, 90 patients were recruited: HC, n = 24, RF, n = 23, CAD, n = 19 and ACS, n = 24. Blood samples were collected by peripheral venipuncture as outlined below, by one individual and all samples were transferred to the laboratory within 10 min. Cohort clinical characteristics are detailed in Supplementary Table 1.

*Platelet isolation from patients.* Whole blood was taken from the antecubital vein using a 21-gauge butterfly needle into a 50 mL syringe containing acidified citrate dextrose (ACD; 85 mM trisodium citrate, 65 mM citric acid, 100 mM glucose, pH 5.0) at a ratio of 8.1 parts whole blood to 1.9 parts ACD, as described previously (4), and centrifuged at 250 × *g* for 10 min at 20 °C. The platelet-rich plasma was collected and centrifuged at 1000 × *g* for 8 min at 20 °C. Platelet poor plasma was removed and retained for extracellular vesicle isolation. The platelet pellet was resuspended in Tyrode's buffer (134 mM NaCl, 12 mM NaHCO_3_, 2.9 mM KCl, 0.34 mM Na_2_HPO_4_, 1.0 mM MgCl_2_, 10 mM HEPES, 5 mM glucose, pH 7.4) containing ACD (9:1, v/v). The platelets were washed by centrifuging at 1000 × *g* for 8 min at 20 °C then resuspended in Tyrode's buffer at a concentration of 2 × 10^8^/mL. Platelets were activated at 37 °C in the presence of 1 mM CaCl_2_, 0.2 U/mL bovine thrombin (Sigma Aldrich) for 30 min with occasional inversion.

*Extracellular vesicle (EV) isolation from patients.* Methods were adapted from recent literature and guidelines (7, 8). Platelet poor plasma generated above was centrifuged at 1000 x *g* for 10 min at 20 °C to generate platelet-free plasma (PFP). 1 mL of this was snap frozen on dry ice and stored at -80 °C for EV quantification later, see below). For each donor plasma, 6 x 1 mL PFP aliquots were centrifuged at 16,000 x *g* for 30 min at 20 °C. 750 μL was removed from each aliquot, and 750 μL of modified Tyrode’s buffer was added to the pellet, which was gently resuspended using a pipette. Following a second centrifugation at 16,000 x *g* for 30 min at 20 °C, 950 μL was removed. 50 μL modified Tyrode’s buffer was added to the pellet to gently resuspend and recover the EV-rich fraction. The six EV fractions were pooled to generate one isolate (300 μL) per donor. Of this, 167 μL was used for lipid extraction (Equivalent to EV isolated from 3.3 mL PFP).

*Extracellular vesicle (EV) quantification.* EV quantification was performed by thawing one aliquot of PFP per patient, of which 500 μL was passed through size-exclusion chromatography iZON qEV columns (Izon Science Ltd, UK) to recover particles and vesicles between 70 nm to 1000 nm in diameter. The eluting EV-rich fractions were collected and analyzed using nanoparticle tracking on a NanoSight 300 (Malvern, UK) equipped with a sensitive sCMOS camera and a 488 nm blue laser, to generate an EV count and size distribution for all participants’ plasmas.

*Leukocyte isolation from patients.* Leukocytes were isolated from 20 mL citrate-anticoagulated whole blood as described previously (4). Briefly, 20 mL of blood was mixed with 4 mL of 2 % citrate and 4 mL of Hetasep (Stem Cell Technologies) and allowed to sediment for 45 minutes. The upper plasma layer was recovered and centrifuged at 250 x *g* for 10 min at 4 °C. The pellet was resuspended in ice-cold 0.4 % trisodium citrate/PBS and centrifuged at 250 x *g* for 5 min at 4 °C. Erythrocytes were removed by hypotonic lysis (0.2 % hypotonic saline). Leukocytes were resuspended in Krebs buffer (100 mM NaCl, 48 mM HEPES, 5 mM KCl, 1 mM sodium dihydrogen orthophosphate dihydrate and 2 mM glucose) at 4 x 10^6^/mL.

*Lipid extraction and LC-MS/MS analysis from whole blood, platelets, leukocytes, and EV.*

200 µL aliquots of whole blood from healthy volunteers were mixed with 800 μL PBS containing 100 µM DTPA, 100 µM BHT and 75 µM acetaminophen, and vortexed for 30 s. Lipids were extracted from samples using hexane:isopropanol:1 M acetic acid (30:20:2) as previously described (9), with 20 μL SPLASH mix as an internal standard (IS) (5 µg/mL PS 15:0/18:1[D7], from Avanti Polar Lipids (Alabama, USA)). Following drying by RapidVap (Labconco, Missouri, USA), lipids were reconstituted in 200 or 400 µL methanol and stored at -80 °C until use. Extracts were diluted with methanol in some experiments. Biotinylated lipids from derivatized platelets were extracted using a modified Bligh and Dyer method as described previously (9). 20 ng biotinylated PS 14:0/14:0 (PS 28:0 Biotin) was added as an IS during extraction. Following drying, lipids were reconstituted in 100 µL methanol and stored at -80 °C until use. Lipids were detected, analyzed, and quantified using several LC-MS/MS approaches, detailed below.

Lipids from clinical cohort samples (platelets: 2x10^8^, leukocytes: 4x10^6^, EVs: from 3.3 mL PFP) were extracted using hexane:isopropanol:1 M acetic acid as described previously (9). Following LC-MS/MS analysis in a previous study (3), the stored extracts were retrieved and added to an equal volume of methanol containing SPLASH mix (PS 15:0/18:1[D7]: 0.05 ng/µL, final conc.), then analyzed using LC-MS/MS for PS and PT as described below. Since the IS was added after extraction, we have not reported fully quantified values for samples from the cohort, instead calculating analyte (cps)/internal standard (cps) values.

*Targeted LC-MS/MS of native PS and PT.* Following lipid extraction as described above, PS and PT were analyzed using hydrophilic interaction chromatography (HILIC) LC-MS/MS, on a 6500 QTRAP mass spectrometer (Sciex, Massachusetts, USA) coupled with a Nexera XR HPLC system (Shimadzu, Kyoto, Japan). Lipids were separated on an Xbridge Amide column (3.5 µm, 4.6 x 150 mm, Waters Corporation, Massachusetts, USA) using solvent A (50:50 water/acetonitrile, 10 mM ammonium formate, 0.1% formic acid) and solvent B (5:95 water/acetonitrile, 10 mM ammonium formate, 0.1% formic acid). Gradient: 0–10 min 100%–80% B; 10–25 min 80%–20%B; 25–26 min 20%–100% B; 26–30 min 100% B. Flow rate was 0.6 mL/min, and the column temperature was maintained at 45 °C. The eluate was introduced into the ESI(-) source for MS analysis, except between 0 – 9 min and 16 – 30 min where the eluate was diverted to waste. Neutral loss scans in negative ion mode for precursors losing 101 Da (PT) or 87 Da (PS) were carried out with the following instrument (6500 QTRAP) parameters: scan speed: 1000 Da/s, scan range: m/z 600–900, declustering potential (DP): -60 V, collision energy (CE): -36 V, cell exit potential (CXP): -32 V, entrance potential (EP): -10 V. Fatty acid compositions of PT and PS species were determined by MS^3^ scans with the following parameters: scan speed: 1000 or 10,000 Da/s, first precursor: [M-H]^-^, second precursor: [M-H-101]^-^, scan range: m/z 100–900, CE ramp: -36 to -46 V, DP: -60 V, EP: -10 V. Lipid species were measured using multiple reaction monitoring (MRM), targeting precursor to product ion transitions (Supplementary Table 2)**.** Data were collected using Analyst Software (version 1.7, Sciex). Peaks (criteria: 5:1 signal to noise (S:N) ratio, with at least 6 points across) were manually integrated using MultiQuant Software (Sciex). Ratio (A/IS, cps) values for each analyte were calculated by dividing the area of the analyte by the area of the internal standard (IS). In some experiments, ratios of areas of analytes to internal standard (A/IS) were used to quantify PT and PS based on six-point calibration curves (Supplementary Figure 20). SPLASH mix (containing PS 15:0/18:1[D7] at 5 ng/µL) was used as IS in some samples and calibration curves. To construct the calibration curves, solutions of lipid standards (PT 16:0/18:1(9Z) and PS 18:0/20:4) were prepared by serial dilution (1/2 or 1/5) in methanol, and 10 µL SPLASH mix was added to each, giving final volumes of 200 µL containing PS 15:0/18:1[D7] at 0.25 ng/µL and the lipid standards at six different concentrations (PT: from 1.969 to 0.003938 ng/µL, PS: from 1.0875 to 0.002175 ng/µL). Samples were analyzed using HILIC LC-MS/MS as described above with MRM-based detection and injection volume of 10 µL.

The HILIC-LC method was also used to partially purify PS and PT by diverting the LC output into a collection tube at the appropriate elution time. The collected fraction (PS-PT fraction) was then dried using nitrogen, reconstituted in methanol (same volume injected for purification), and stored at -80 °C until use. Standards for PT 16:0/18:1(9Z) and PT 18:0/18:1(9Z) were synthesized as described previously (10). Since only headgroups are used for MRM methods, and in some cases, the lipids may include more than one molecular species, lipids detected in biological samples are labelled using the LIPID MAPS shorthand nomenclature, as outlined here (11).

*Analysis of externalized PS and PT.* Biotinylated PS 14:0/14:0 was synthesized as described previously (12). Resting or thrombin-activated washed platelets (4 x 10^7^, 200 µL), prepared as described above, were incubated with either sulfo-NHS-biotin (11 mM in PBS) or NHS-biotin (20 mM in DMSO, both from Thermo Fisher Scientific) for 10 min at 21 °C, then the reaction was quenched using L-lysine (50 mM) for a further 10 min at 21 °C, for samples with sulfo-NHS-biotin only. PBS was added to samples to make 1 mL prior to lipid extraction. Biotinylated PS and PT were analyzed using reversed phase (RP)-LC-MS/MS, on a 6500 QTRAP mass spectrometer (Sciex) coupled with a Nexera XR HPLC system (Shimadzu). Lipids were separated on an Ascentis C18 column (5 µm, 2.1 x 150 mm, Sigma Aldrich) using isocratic elution with methanol, 0.2 % (w/v) ammonium acetate (2.6 mM) for 25 min. Flowrate was 0.4 mL/min, and the column temperature was maintained at 22 °C. The eluate was introduced into the ESI(-) source, and MS detection was done by MRM (Supplementary Table 3). The correct biotinylated PT peaks were confirmed using MS^2^ scans (Supplementary Figures 12,13) with the following parameters: scan speed: 1000 Da/s, scan range: m/z 100–1000, CE: -48 V, DP: -300 V, EP: -10 V. The following instrument (6500 QTRAP) parameters were used: Source/gas: curtain gas flow (CUR): 35, temperature (TEM): 500, ion source gas 1 (GS1): 40, ion source gas 2 (GS2): 30, and ion spray voltage (IS): -4500 V. Detector: single channel electron multiplier (CEM): 2200.

Peaks (criteria: 5:1 signal to noise (S:N) ratio, with at least 6 points across) were manually integrated using MultiQuant Software (Sciex). Ratio (A/IS, cps) values for each analyte were calculated by dividing the area of the analyte by the area of the internal standard (IS). Biotinylated PS 14:0/14:0 (PS 28:0 Biotin) was used as an IS for both biotinylated PS and PT since a biotinylated PT standard was not available.

*High resolution mass spectrometry of PT.* High mass accuracy measurements were carried out using a SYNAPT XS Q-TOF mass spectrometer (Waters Corporation) coupled with an Acquity UPLC system (Waters Corporation), using the HILIC LC method detailed above. MS^2^ scans of PT species were collected in the negative polarity using the resolution mode and the following settings: scan time: 1 s, range: m/z 50–1000, CE: -31 V. ESI settings: capillary voltage: 2400 V, cone voltage: 25 V, source offset: 4 V, source temperature: 120 °C, desolvation temperature: 500 °C, cone gas: 20 L/h, desolvation gas: 1000 L/h, nebulizer: 6.5 Bar. Leucine enkephalin (554.2615 Da) was used as a LockSpray calibrant for mass correction and was sprayed at 10 s intervals with a scan time of 1 s. Data were collected using MassLynx (version 4.2, Waters Corporation). MS^2^ scans of PT standards (PT 18:0/18:1(9Z) and PT 16:0/18:1(9Z)) were collected using standard solutions (0.5 ng/µL in methanol) and the method described above (Supplementary Figure 9).

*Targeted LC-MS/MS of amino acids.* 1 μg PT 16:0/18:1(9Z) standard or 100 μL of purified PS-PT fraction were hydrolyzed using 200 μL (final volume) 3 M HCl in methanol/water (1:1) and incubation at 100 °C for 1 h. The hydrolysate was dried using nitrogen gas and reconstituted in 200 μL (standard) or 80 μL (sample) acetonitrile/water (1:1) + 0.1% formic acid for analysis. Standard solutions of L-serine and L-threonine (0.1 ng/μL) were prepared in acetonitrile/water (1:1) + 0.1% formic acid to test chromatographic separation and compare retention times with samples. HILIC LC-MS/MS analysis of amino acids was carried out on a 4000 QTRAP mass spectrometer (Sciex) coupled with a Nexera XR HPLC system (Shimadzu). Amino acids were separated on an Xbridge Amide column (3.5 µm, 4.6 x 150 mm, Waters Corporation) using solvent A (50:50 water/acetonitrile, 10 mM ammonium formate, 0.1% formic acid) and solvent B (5:95 water/acetonitrile, 10 mM ammonium formate, 0.1% formic acid). Gradient: 0–15 min 100%–0% B; 15–16 min 0%–100% B; 16–20 min 100% B. Flow rate was 0.6 mL/min, and the column temperature was maintained at 45 °C. The eluate was introduced into the ESI(+) source, and MS detection was done by MRM. (Supplementary Table 4). Injection volume was 20 µL. The following instrument (4000 QTRAP) parameters were used: Source/gas: CUR: 20, TEM: 500, GS1: 40, GS2: 30, and IS: 4500 V. Detector: CEM: 2300.

*Preparation of liposomes by membrane extrusion.* Lipids (PC 16:0/18:1(9Z), PC 18:0/18:0, PS 16:0/18:1(9Z), PS 18:0/18:1(9Z), PE 18:0/18:1(9Z)) were from Avanti Lipids (Alabama). Liposomes were made using PC 18:0/18:0, PE 18:0/18:1(9Z), PS 18:0/18:1(9Z), and PT 18:0/18:1(9Z) in Buffer (A: 10 mM HEPES, 10 mM NaCl, pH 7.35, or B: 20 mM HEPES, 140 mM NaCl, pH 7.35), followed by membrane extrusion. For this, lipids in organic solvents were mixed in desired mol ratios then evaporated using a RapidVap (Labconco). Dry lipid films were hydrated with buffer, vortexed for 30 s, then subjected to 10 freeze-thaw cycles (30 s freezing and 30 s thawing) with agitation using liquid nitrogen and a water bath at 60 °C. For tissue factor (TF)-bearing liposomes, TF (full length, Haematologic Technologies (Vermont, USA)) was included in the buffer, and the thawing step was carried out at 37° C instead of 60 °C. Liposomes were extruded 19 times through a 100-nm membrane (LiposoFast polycarbonate membranes) from Avestin (Ottawa, Canada). All liposome compositions are in Supplementary Table 5.

*Coagulation assays:*

Human FVa was from Haematologic Technologies (Vermont, USA). Human prothrombin (FII), thrombin (FIIa), FXa, FX, FVIIa, and were from Enzyme Research Laboratories (Indiana, USA). FXa chromogenic substrate S-2765 was from Quadratech Diagnostics (Cooksbridge, UK). Thrombin calibrator (T-cal) was from Stago (Asnières-sur-Seine, France). Thrombin fluorogenic substrate (Z-Gly-Gly-Arg-AMC) was from Bachem (Bubendorf, Switzerland).

1. *Prothrombinase assay**.* In a 96-well plate, liposomes (25 μM total lipid, in buffer B: 20 mM HEPES and 140 mM NaCl, pH 7.35) were mixed with coagulation buffer (20 mM Tris, 150 mM NaCl, and 0.05% (w/v) bovine serum albumin (BSA), pH 7.35) containing (final concentrations) FII (200 nM), FVa (3.0 nM), FXa (10 nM), and CaCl_2_ (1 mM), in final volume 40 µL, then incubated at 21 °C for 5 min. The reaction was stopped by adding EDTA (10 µL, final: 7 mM). Next, chromogenic substrate S-2238 (final: 0.8 mM, Pefachrome TH 8198, Enzyme Research Laboratories (Indiana, USA)) was added and absorbance at 405 nm was monitored for 50 minutes using a CLARIOstar Plus microplate reader (BMG Labtech, Ortenberg, Germany). Relative prothrombinase activity values were calculated by determining the slope of the absorbance curves from 0 – 10 min. All liposome preparations were made in triplicate, and each was tested 2-3 times.
2. *Calibrated automated thrombinography (CAT).* In a 96-well plate, TF-liposomes (10 µM total lipid, 50 pM TF, in Buffer B) or T-cal (calibrator) were mixed (1:4) with pooled CTI-PPP, in final volume 100 µL, and incubated at 37 °C for 10 min. After incubation, 20 µL Buffer C (20 mM HEPES, 0.02% (w/v) sodium azide, and 0.6 % (w/v) BSA, pH 7.35) containing calcium (final: 16.7 mM) and fluorogenic thrombin substrate (final: 0.42 mM) was added to samples and fluorescence was measured for 60 min using a Fluoroskan Ascent microplate fluorometer (Thermo Fischer Scietific). Peak thrombin and lag time were automatically calculated by the Thrombinoscope software (Stago) using the calibrator reference curve, based on a previously described method (13). All liposomes formulations were made and tested in triplicate.
3. *Extrinsic tenase assay.* In a 96-well plate, TF-liposomes (25 μM total lipid, 1 nM TF, in buffer B) were mixed with coagulation buffer containing (final concentrations) FX (500 nM), FVIIa (25 nM), and CaCl_2_ (2.5 mM), then immediately chromogenic FXa substrate S-2765 (final: 0.93 mM) was added to each well and absorbance at 405 nm was monitored for 40 minutes using a CLARIOstar Plus microplate reader (BMG Labtech). Relative tenase activity values were calculated by determining the slope of the absorbance curves from 0 – 10 min. All liposome formulations were tested in triplicate, with each individual formulation being tested 2-3 times.

*Preparation of nanodiscs and surface plasmon resonance.* Proteins used in surface plasmon resonance (human FVa, FX, prothrombin, and bovine lactadherin) were from Haematologic Technologies (Vermont, USA). Nanodiscs of 10 nm diameter were prepared with MSP1D1 membrane scaffold protein by self-assembly reactions followed by gel filtration as described previously, using various ratios (mol %) of PC 16:0/18:1(9Z), PS 16:0/18:1(9Z) and PT 16:0/18:1(9Z) (14). Binding affinities for clotting proteins factor X (FX), prothrombin, factor Va (FVa) and lactadherin to various nanodiscs were measured using surface plasmon resonance (SPR) using Biacore T-200 instrument (Cytiva, formerly GE Healthcare, Massachusetts, USA). Nanodiscs (ND) were immobilized over Ni-NTA Series S Sensor Chip at 25 µL/min flow rate in loading buffer (20 mM Tris, 100 mM NaCl, 0.02% (w/v) sodium azide) for 5 sec to achieve a net change of ~700–1200 Response Units (RU_ND_). After immobilization, proteins (FX, prothrombin, FVa, or lactadherin) at increasing concentrations were flowed over the immobilized nanodiscs at 30 µL/min in loading buffer containing 5 mM calcium and 0.2% (w/v) BSA. The binding isotherms were generated as described previously (15). The net change in the response units observed upon protein binding to nanodiscs were divided by RU_ND._ Background binding of proteins to 100% PC was subtracted from the ratio RU _(protein= FX, FVa, prothrombin, & lactadherin)_ / RU_ND_ and fitted against the increasing concentrations of proteins using the following equation:

RU_protein_/RU_ND_= (B_max_ x X) / (K_d_ + X)………………………..…[1]

Where B_max_ is the maximal binding of protein to nanodisc at saturation, X is the concentration of the protein and K_d_ is the binding affinity.

*Comment on calcium binding assay*

During revision it was noted that absorbance of PC/PE liposomes was higher than either PC/PE/PS or PC/PE/PT. Our method was based on that used by Martín-Molina and Sinn (16, 17). In Sinn et al, only one composition of membrane was used. Here, DOPS/DOPC (20% PS) had a baseline absorbance of around 0.5, at 350 nm. In Martín-Molina, while different lipid membranes were compared in a titration with calcium, absorbance changes at 400 nm are shown relative to the calcium-free control. Baseline absorbance values were not provided Sinn et al, and so we could not compare different compositions. Thus, we are unable to compare our data with these literature values.

We expect that the absorbance of liposome suspensions at 400 nm may be influenced by composition having an impact on light scattering properties. Notably, PC/PE/PT and PC/PE/PS absorbed light similarly in the absence of calcium, and their response to added calcium was similar.

Different hypotheses exist for why liposomes absorb/scatter more light when binding calcium, such as aggregation or transition of lipids from liquid phase to a gel phase due to their interaction with calcium ions (leading to reduced light transmission). In support of the latter, calcium ions increase the transition temperatures of lipids, as evidenced by differential scanning calorimetry measurements (shown in Sinn et al). In our studies, liposomes were always prepared fresh and tested on the same day, to reduce variability.

**Supplementary Table 1. Baseline clinical characteristics of patients recruited in the clinical cohort.** (WCC: white cell count, RBC: red blood cell count, P2Y12 inhibitors: clopidogrel, prasugrel or ticagrelor, CKD: chronic kidney disease, SD: standard deviation, p-value tests: Fisher exact (categorical) or Kruskal-Wallis (continuous), p-value comparators: all clinical groups (age, gender) or all except HC for other variables). Data reproduced from Protty et al. (3).


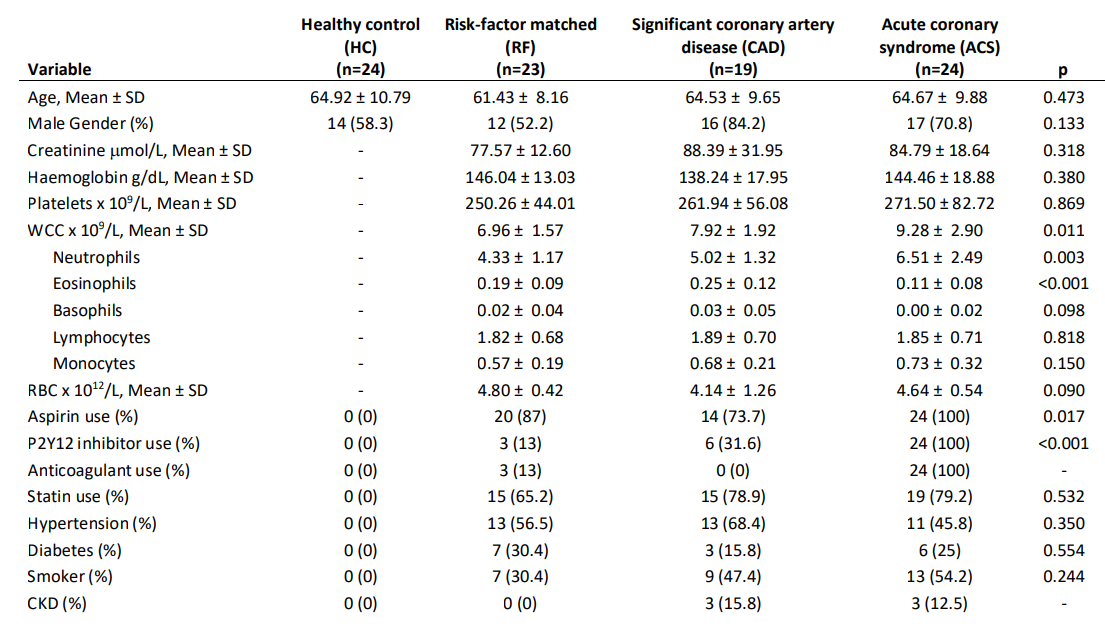


**Supplementary Table 2. Negative ion mode MRM transitions used for PS and PT species.** The following parameters were used for all analytes: Dwell time: 100 ms, declustering potential: -60 V, collision energy: -36 V, cell exit potential: -32 V.

| **Analyte** | **Q1 (*m/z*)** | **Q3 (*m/z*)** |
| --- | --- | --- |
| PS 33:1[D7] (internal standard) | 753.5 | 666.5 |
| PS 34:1 | 760.5 | 673.5 |
| PS 36:1 | 788.6 | 701.6 |
| PS 36:2 | 786.5 | 699.5 |
| PS 38:3 | 812.5 | 725.5 |
| PS 38:4 | 810.6 | 723.6 |
| PS 38:5 | 808.5 | 721.5 |
| PS 40:3 | 840.6 | 753.6 |
| PS 40:4 | 838.7 | 751.7 |
| PS 40:5 | 836.7 | 749.7 |
| PS 40:6 | 834.6 | 747.6 |
| PT 34:1 | 774.5 | 673.5 |
| PT 36:1 | 802.6 | 701.6 |
| PT 36:2 | 800.5 | 699.5 |
| PT 38:3 | 826.5 | 725.5 |
| PT 38:4 | 824.6 | 723.6 |
| PT 38:5 | 822.5 | 721.5 |
| PT 40:3 | 854.6 | 753.6 |
| PT 40:4 | 852.7 | 751.7 |
| PT 40:5 | 850.7 | 749.7 |
| PT 40:6 | 848.6 | 747.6 |

**Supplementary Table 3. Negative ion mode MRM transitions used for biotinylated PS and PT species.**The following parameters were used for all analytes: Dwell time: 100 ms, declustering potential: -300 V, collision energy: -48 V, cell exit potential: -21 V.

| **Analyte** | **Q1 (*m/z*)** | **Q3 (*m/z*)** |
| --- | --- | --- |
| PS 28:0 Biotin (internal standard) | 904.4 | 591.4 |
| PS 36:1 Biotin | 1014.6 | 701.6 |
| PS 36:2 Biotin | 1012.5 | 699.5 |
| PS 38:3 Biotin | 1038.5 | 725.5 |
| PS 38:4 Biotin | 1036.6 | 723.6 |
| PS 40:6 Biotin | 1060.6 | 747.6 |
| PT 36:1 Biotin | 1028.6 | 701.6 |
| PT 36:2 Biotin | 1026.5 | 699.5 |
| PT 38:3 Biotin | 1052.5 | 725.5 |
| PT 38:4 Biotin | 1050.6 | 723.6 |
| PT 40:6 Biotin | 1074.6 | 747.6 |

**Supplementary Table 4. Positive ion mode MRM transitions used for serine and threonine LC-MS/MS analysis.**

| **Analyte** | **Q1 (*m/z*)** | **Q3 (*m/z*)** | **Dwell time (ms)** | **DP (volts)** | **CE (volts)** | **CXP (volts)** |
| --- | --- | --- | --- | --- | --- | --- |
| Serine | 106.0 | 60.0 | 200 | 11 | 15 | 10 |
| Threonine | 120.1 | 74.1 | 200 | 21 | 15 | 4 |

**Supplementary Table 5. Liposome compositions that were used for the different assays.**

Buffer A: 10 mM HEPES, 10 mM NaCl, pH 7.35. Buffer B: 20 mM HEPES, 140 mM NaCl, pH 7.35. TF: tissue factor.

| **Assay** | **[Total Lipid]** | **Composition #** | **PC 18:0/18:0 mol %** | **PE 18:0/18:1(9Z) mol %** | **PS 18:0/18:1(9Z) mol %** | **PT 18:0/18:1(9Z) mol %** | **[TF]** | **Buffer** |
| --- | --- | --- | --- | --- | --- | --- | --- | --- |
| Calcium binding assay | 1 mM | 1 | 60 | 40 | 0 | 0 | none | A |
|  |  | 2 | 60 | 30 | 10 | 0 |  |  |
|  |  | 3 | 60 | 30 | 0 | 10 |  |  |
| Prothrombinase assay | 25 µM | 4 | 60 | 40 | 0 | 0 | none | B |
|  |  | 5 | 60 | 30 | 10 | 0 |  |  |
|  |  | 6 | 60 | 30 | 0 | 10 |  |  |
|  |  | 7 | 59.86 | 30 | 10 | 0.14 |  |  |
|  |  | 8 | 59.72 | 30 | 10 | 0.28 |  |  |
|  |  | 9 | 95 | 0 | 0 | 5 |  |  |
|  |  | 10 | 90 | 0 | 0 | 10 |  |  |
|  |  | 11 | 85 | 0 | 0 | 15 |  |  |
|  |  | 12 | 80 | 0 | 0 | 20 |  |  |
|  |  | 13 | 80 | 20 | 0 | 0 |  |  |
|  |  | 14 | 80 | 0 | 20 | 0 |  |  |
| Calibrated automated thrombinography | 10 µM | 15 | 60 | 40 | 0 | 0 | 50 pM | B |
|  |  | 16 | 60 | 30 | 10 | 0 |  |  |
|  |  | 17 | 60 | 30 | 0 | 10 |  |  |
|  |  | 18 | 59.86 | 30 | 10 | 0.14 |  |  |
|  |  | 19 | 80 | 20 | 0 | 0 |  |  |
|  |  | 20 | 80 | 0 | 20 | 0 |  |  |
|  |  | 21 | 80 | 0 | 0 | 20 |  |  |
| Extrinsic tenase assay | 25 µM | 22 | 80 | 20 | 0 | 0 | 1 nM | B |
|  |  | 23 | 80 | 0 | 20 | 0 |  |  |
|  |  | 24 | 80 | 0 | 0 | 20 |  |  |


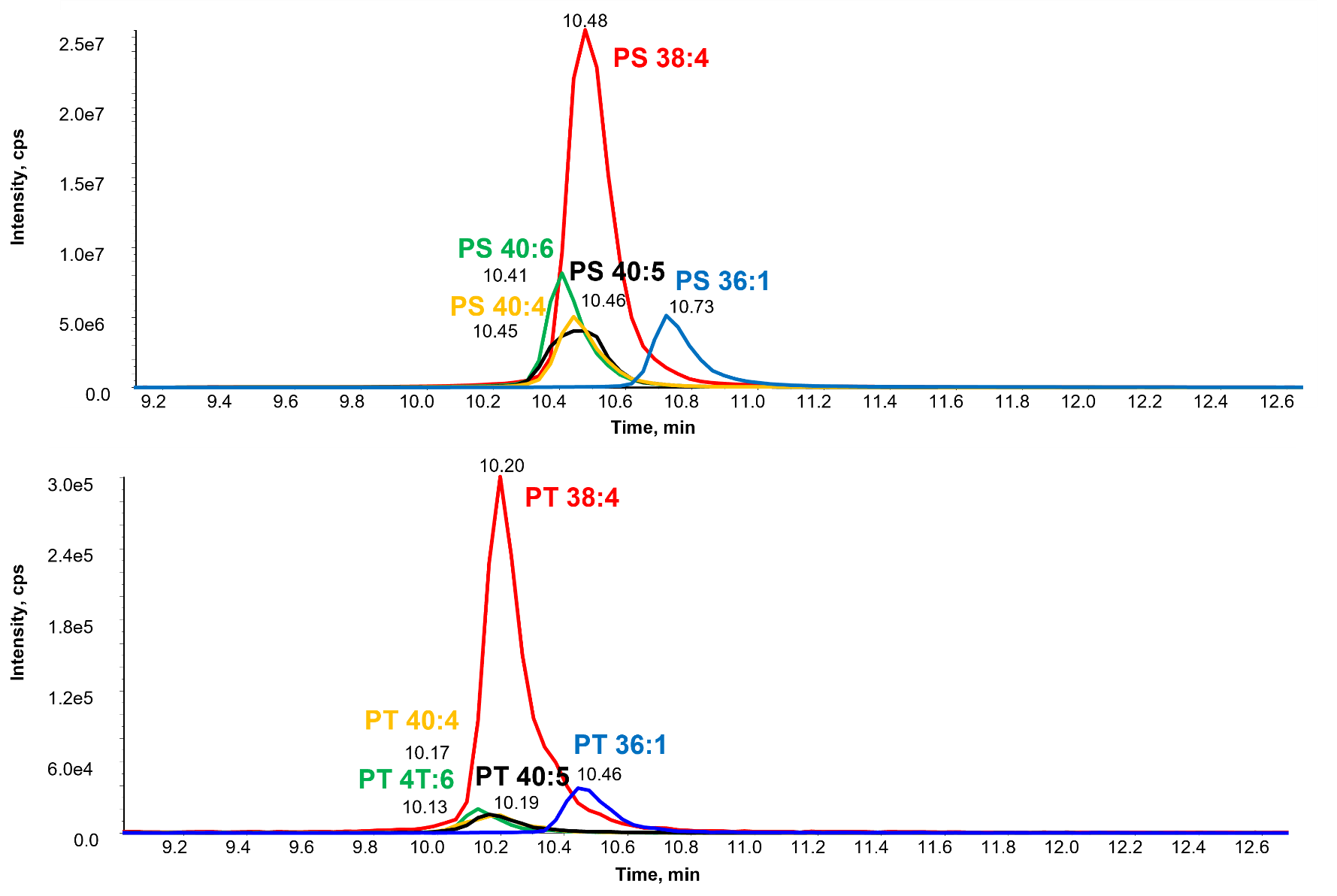


**Supplementary Figure 1. Extracted ion chromatograms of PS and PT species detected in whole blood lipid extract using HILIC LC-MS/MS in the negative ion mode show coelution of PS and PT.**

Lipids in whole blood from healthy volunteers were extracted and analyzed using HILIC LC-MS/MS as described in Supplementary Methods (MRMs in Supplementary Table 2). The PT species elute earlier than the corresponding PS species of the same species level structure (e.g., PT 38:4 and PS 38:4), although there is some small overlap in retention times.


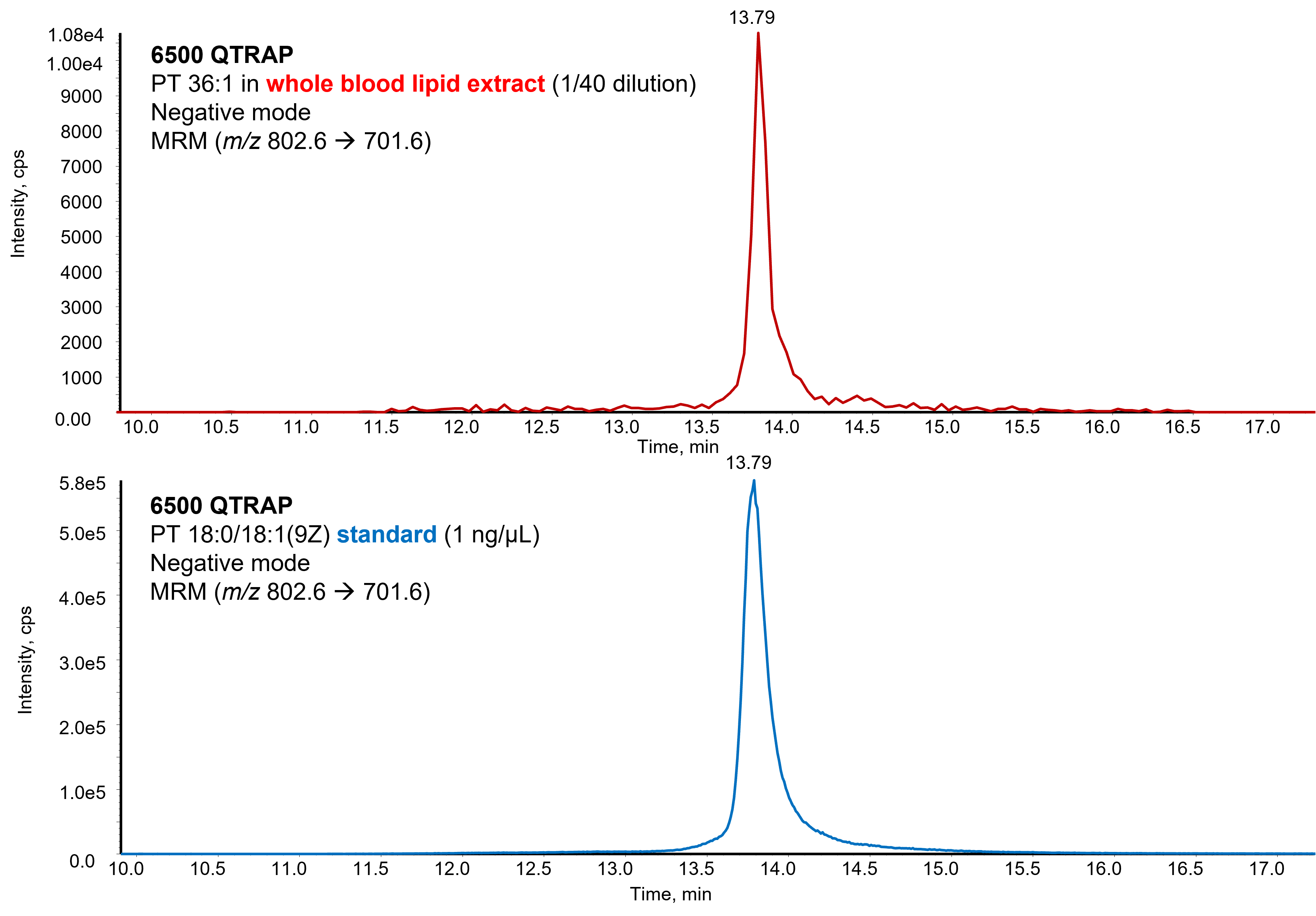


**Supplementary Figure 2. Extracted ion chromatograms of PT 36:1 in whole blood lipid extract (top) and a PT 18:0/18:1(9Z) standard (bottom), analyzed using HILIC LC-MS/MS in the negative ion mode.**

Whole blood lipid extract from a healthy volunteer, prepared as described in Supplementary Methods and diluted 1/40 in methanol, and a PT 18:0/18:1(9Z) standard (1 ng/µL in methanol) were analyzed using HILIC LC-MS/MS as described in Supplementary Methods (MRMs in Supplementary Table 2). PT 36:1 in the blood lipid extract has the same retention time as the synthetic standard. Note, retention time drift between solvent batches and sample batches is common in HILIC. An internal standard (PS 15:0/18:1[D7]) that coelutes with the analytes of interest included in sample analysis enabled this drift to be monitored.


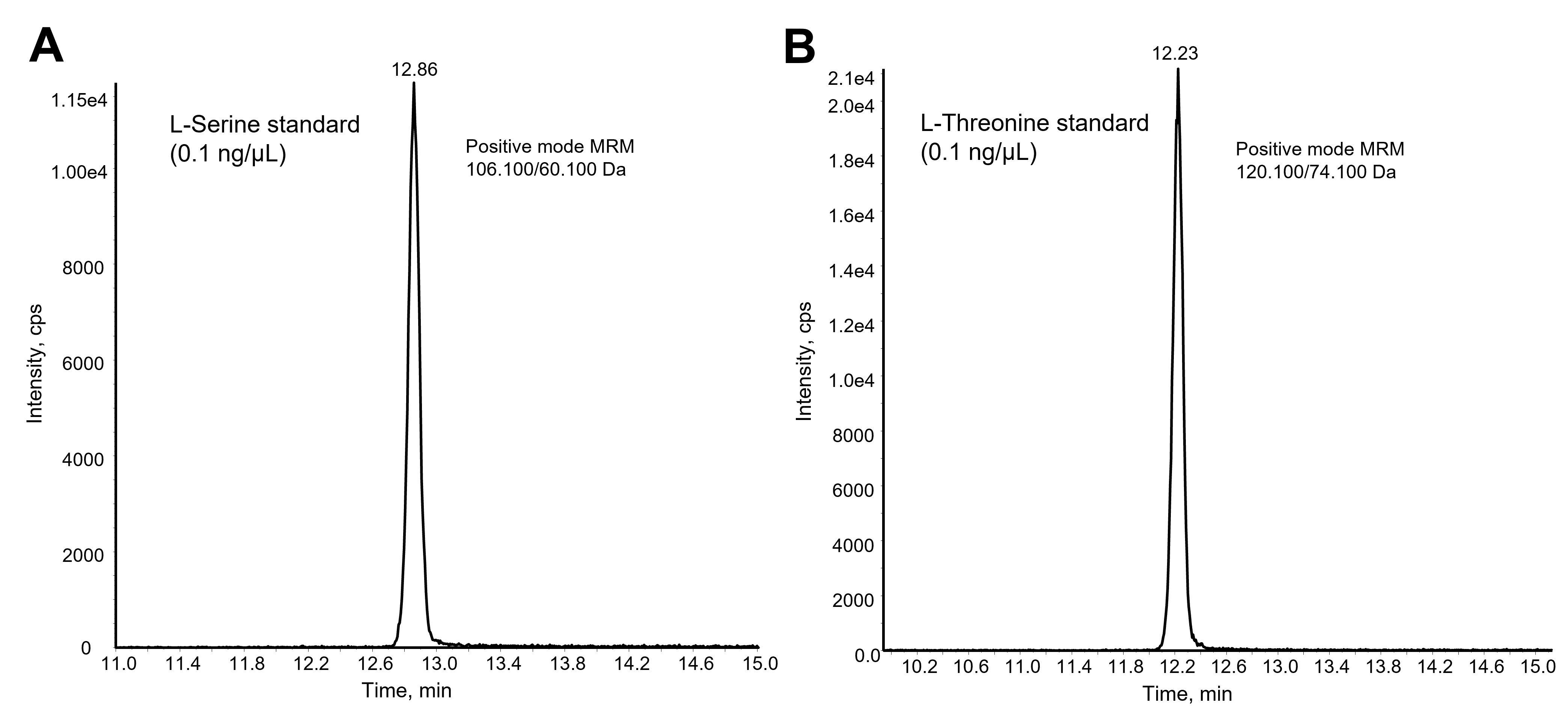


m/z 106.1/60.1

m/z 120.1/74.1

**Supplementary Figure 3. Extracted ion chromatograms of amino acid standards detected using HILIC LC-MS/MS in the positive ion mode.**

L-serine and L-threonine standards (0.1 ng/µL) in acetonitrile/water (1:1) + 0.1% formic acid were analyzed using HILIC LC-MS/MS as described in Supplementary Methods (MRMs in Supplementary Table 4). Retention times of these standards were consistent with serine and threonine in the acid hydrolysate of blood lipid extracts (Figure 1 B, main text).

*m/z* 106.1/60.1, Serine

11.0

12.0

13.0

14.0

Time, min

0.0

1.0e4

2.0e4

3.0e4

Intensity, cps

12.84

*m/z* 120.1/74.1, Threonine

11.0

12.0

13.0

14.0

Time, min

0

500

1000

1500

Intensity, cps

12.25

11.0

12.0

13.0

14.0

Time, min

0

50

100

Intensity, cps

12.84

12.21

*m/z* 106.1/60.1 Serine

*m/z* 120.1/74.1

Threonine

Lipid extract

Purified PS/PT fraction from blood, after hydrolysis

**A**

**C**

**B**

Lipid extract

Purified PS/PT fraction from blood, after hydrolysis

Blank showing level of background signals on LC-MS/MS system

**Supplementary Figure 4. Serine or threonine analysis of lipid extracts prior to hydrolysis of lipid extracts from blood shows background trace levels only.** Lipid extracts were generated as described in methods. They were either analyzed using LC-MS/MS (blue traces) or hydrolyzed as described in methods prior to analysis (red traces). Although a small signal shows up prior to hydrolysis, this is similar to blank signals seen apparently resulting from small levels of carryover that could not be easily eliminated (*Panel C*). *Panel A*: Serine. *Panel B*: Threonine.

8

12

16

Time, min

5.0e4

1.0e5

1.5e5

Intensity, cps

No hydrolysis

After acid hydrolysis

**Supplementary Figure 5. Acid hydrolysis of PT completely removes the threonine headgroup**. 10 ng PT 36:1 was incubated for 1 hr at 100°C in 50:50 methanol:6M HCl, as described in Methods. Following hydrolysis, lipids were analysed using LC-MS/MS as described in Methods. Green trace: control lipid, blue trace: hydrolyzed sample.

774.5/673.5 Da

PT 34:1

9.0

9.5

10.0

10.5

11.0

11.5

12.0

12.5

13.0

13.5

14.0

Time, min

0.0

4000.0

8000.0

1.2e4

1.6e4

2.0e4

2.4e4

Intensity, cps

10.75

802.600/701.600 Da

PT 36:1

9.0

9.4

9.8

10.2

10.6

11.0

11.4

11.8

12.2

12.6

13.0

13.4

13.8

Time, min

0.0

4.0e4

8.0e4

1.2e5

1.6e5

2.0e5

Intensity, cps

10.67

800.500/699.500 Da

PT 36:2

9.0

9.5

10.0

10.5

11.0

11.5

12.0

12.5

13.0

13.5

14.0

Time, min

0.0

4000.0

8000.0

1.2e4

1.6e4

2.0e4

Intensity, cps

10.64

822.500/721.500 Da

PT 38:5

9.0

9.4

9.8

10.2

10.6

11.0

11.4

11.8

12.2

12.6

13.0

13.4

13.8

Time, min

0

1000

2000

3000

4000

5000

6000

7000

Intensity, cps

10.52

848.600/747.600 Da

PT 40:6

9.0

9.5

10.0

10.5

11.0

11.5

12.0

12.5

13.0

13.5

14.0

Time, min

0

1000

2000

3000

4000

5000

Intensity, cps

10.47

826.500/725.500 Da

PT 38:3

9.0

9.4

9.8

10.2

10.6

11.0

11.4

11.8

12.2

12.6

13.0

13.4

13.8

Time, min

0.0

4000.0

8000.0

1.2e4

1.6e4

2.0e4

2.4e4

Intensity, cps

10.52

850.700/749.700 Da

PT 40:5

9.0

9.5

10.0

10.5

11.0

11.5

12.0

12.5

13.0

13.5

14.0

Time, min

0

800

1600

2400

3200

4000

4730

Intensity, cps

10.48

852.700/751.700 Da

PT 40:4

9.0

9.5

10.0

10.5

11.0

11.5

12.0

12.5

13.0

13.5

14.0

Time, min

0

1000

2000

3000

4000

5000

6000

Intensity, cps

10.50

854.600/753.600 Da

PT 40:3

9.0

9.5

10.0

10.5

11.0

11.5

12.0

12.5

13.0

13.5

14.0

Time, min

0

200

400

600

800

1000

1200

1400

1600

Intensity, cps

10.49

**Supplementary Figure 6. Representative chromatograms for PT species measured using HILIC LC-MS/MS in the negative ion mode (MRMs in Supplementary Table 2).** Samples are from blood or cell extracts and data is acquired using a 6500 QTRAP, as outlined in Methods.

-MS3 (802.60),(701.60):

100

150

200

250

300

350

400

450

500

550

600

650

700

750

800

850

900

0.0

1.0e4

2.0e4

3.0e4

4.0e4

5.0e4

6.0e4

283.4

437.3

419.3

281.4

PT 36:1 🡪 PT 18:0_18:1

**m/z, Da**

**Intensity, cps**

-MS3 (774.50),(673.50)

100

150

200

250

300

350

400

450

500

550

600

650

700

750

800

850

900

**m/z, Da**

0

400

800

1200

1600

2000

2400

2800

3200

**Intensity, cps**

255.4

281.5

409.4

391.3

417.5

PT 34:1 🡪 PT 16:0_18:1

-MS3 (800.50),(699.50)

100

150

200

250

300

350

400

450

500

550

600

650

700

750

800

850

900

0.00

2000.00

4000.00

6000.00

8000.00

1.00e4

281.4

417.2

435.4

283.4

419.2

437.2

279.4

PT 36:2 🡪 PT 18:1_18:1
& PT 18:0_18:2

**m/z, Da**

**Intensity, cps**

-MS3 (826.50),(725.50)

100

150

200

250

300

350

400

450

500

550

600

650

700

750

800

850

900

0

2000

4000

6000

283.3

437.3

419.3

305.3

459.2

PT 38:3 🡪 PT 18:0_20:3

**m/z, Da**

**Intensity, cps**

-MS3 (822.50),(721.50)

100

150

200

250

300

350

400

450

500

550

600

650

700

750

800

850

900

0

2000

4000

6000

281.4

435.2

417.4

303.5

439.4

PT 38:5 🡪 PT 18:1_20:4

**m/z, Da**

**Intensity, cps**

-MS3 (848.60),(747.60)

100

150

200

250

300

350

400

450

500

550

600

650

700

750

800

850

900

0

1000

2000

3000

4000

283.4

437.5

419.3

327.4

PT 40:6 🡪 PT 18:0_22:6

**m/z, Da**

**Intensity, cps**

-MS3 (850.60),(749.60)

100

150

200

250

300

350

400

450

500

550

600

650

700

750

800

850

900

0

1000

2000

3000

283.4

309.5

419.2

463.3

445.2

329.3

303.5

**m/z, Da**

**Intensity, cps**

PT 40:5 🡪 PT 18:0_22:5 & PT 20:1_20:4

PT 40:4 🡪 PT 18:0_22:4 & PT 20:0_20:4

-MS3 (852.60),(751.60)

100

150

200

250

300

350

400

450

500

550

600

650

700

750

800

850

900

0

1000

2000

3000

283.4

311.4

437.5

447.4

331.4

303.5

419.2

**m/z, Da**

**Intensity, cps**

**Supplementary Figure 7. Representative MS^3^ for PT species measured using HILIC LC-MS/MS in the negative ion mode from blood or cell extracts.** EPI spectra were acquired during elution of lipids as labelled, on a 6500 QTRAP. For one lipid, PT 40:3, definitive MS^3^ spectra could not be obtained since the level of the lipid was relatively low, however the MRM matches the expected retention time for this lipid.

802.6/701.6, PT 36:1

9.0

10.0

11.0

12.0

13.0

14.0

Time, min

0.0

1.0e5

2.0e5

3.0e5

4.0e5

5.0e5

Intensity, cps

11.22

774.5/673.5, PT 34:1

9.0

10.0

11.0

12.0

13.0

14.0

Time, min

0.00

2.00e4

4.00e4

6.00e4

8.00e4

1.00e5

Intensity, cps

11.34

753.5/666.5, PS 33:1[D7]

9.0

10.0

11.0

12.0

13.0

14.0

Time, min

0.0

2.0e4

4.0e4

6.0e4

8.0e4

Intensity, cps

11.81

**A**

**C**

**B**

Spiked

Not spiked

Spiked

Not spiked

Spiked

Not spiked

**Supplementary Figure 8. Spiking lipid extracts confirms the presence of PT 34:1 and PT 36:1 in platelets**. A platelet lipid extract (basal) was spiked with 2.5 ng/50 μl sample prior to LC-MS/MS analysis as described in Methods. *Panel A*: PT 34:1, *Panel B:* PT 36:1, *Panel C*: Internal standard PS 33:1[D7] Red trace: sample without added standards. Blue trace: sample with added standard. Note a small shift in retention time for the lipids on the HILIC column, which is confirmed by showing the internal standard, PS 33:1[D7].


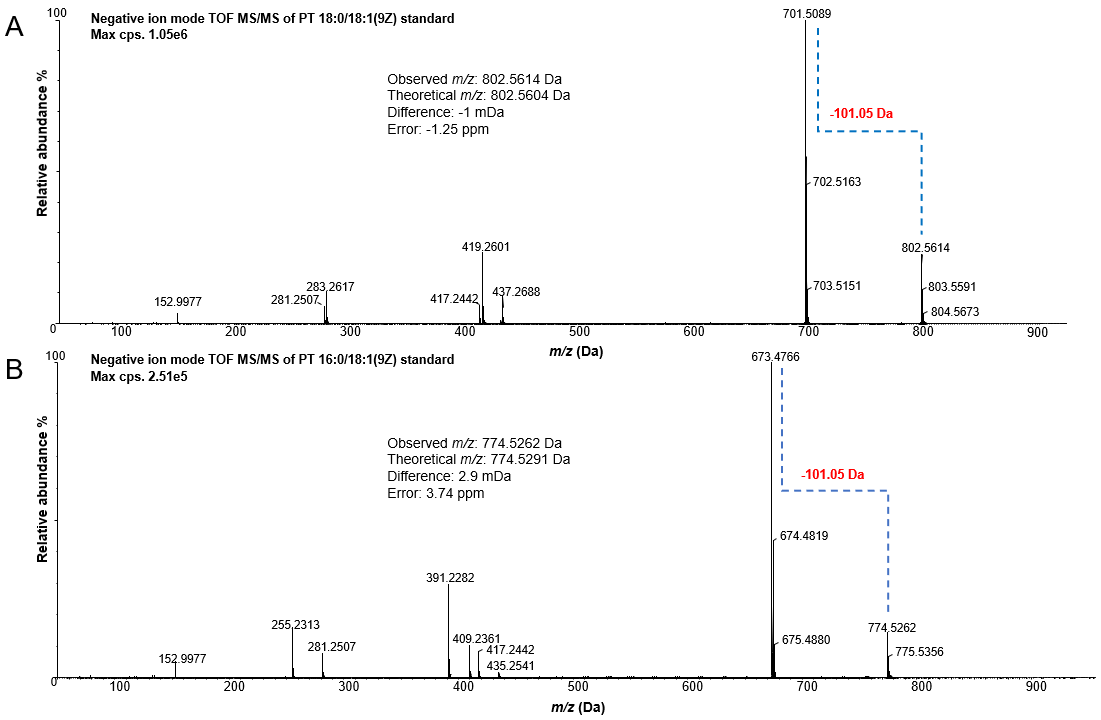


**Supplementary Figure 9. High resolution MS/MS scans of PT standards in the negative ion mode.**

Standard solutions of PT 18:0/18:1(9Z) and PT 16:0/18:1(9Z) (0.5 ng/µL in methanol) were analyzed using HILIC LC-MS/MS on the Q-TOF system as described in Supplementary Methods. (A) MS^2^ scan of PT 18:0/18:1(9Z) (*m/z* 802.5614) acquired at 12.8 min, showing the neutral loss of 101.05 Da and the FA composition: FA 18:0 (*m/z* 283.2617) and FA 18:1 (*m/z* 281.2507). (B) MS^2^ of PT 16:0/18:1(9Z) (*m/z* 774.5262) acquired at 12.9 min, showing the neutral loss of 101.05 Da and the FA composition: FA 16:0 (*m/z* 255.2313) and FA 18:1 (*m/z* 281.2507).

**Supplementary Figure 10. Comparison of standards using serial dilutions shows that PT detection is only slightly more sensitive than PS using HILIC LC/MS/MS, for the same molecular species.** PT 18:0/18:1 and PS 18:0/18:1 were serially diluted to inject from 0.1 – 5 ng on column, and detected as outlined in Methods (n = 2-3 injections per standard). Slopes are calculated by Excel to be relatively similar at 2E6, although manual calculation of values shows that PT detection is around 30% more sensitive than PS. This is not considered to be a major difference, and allows a rough visual comparison of data in Supplementary Figure 12.


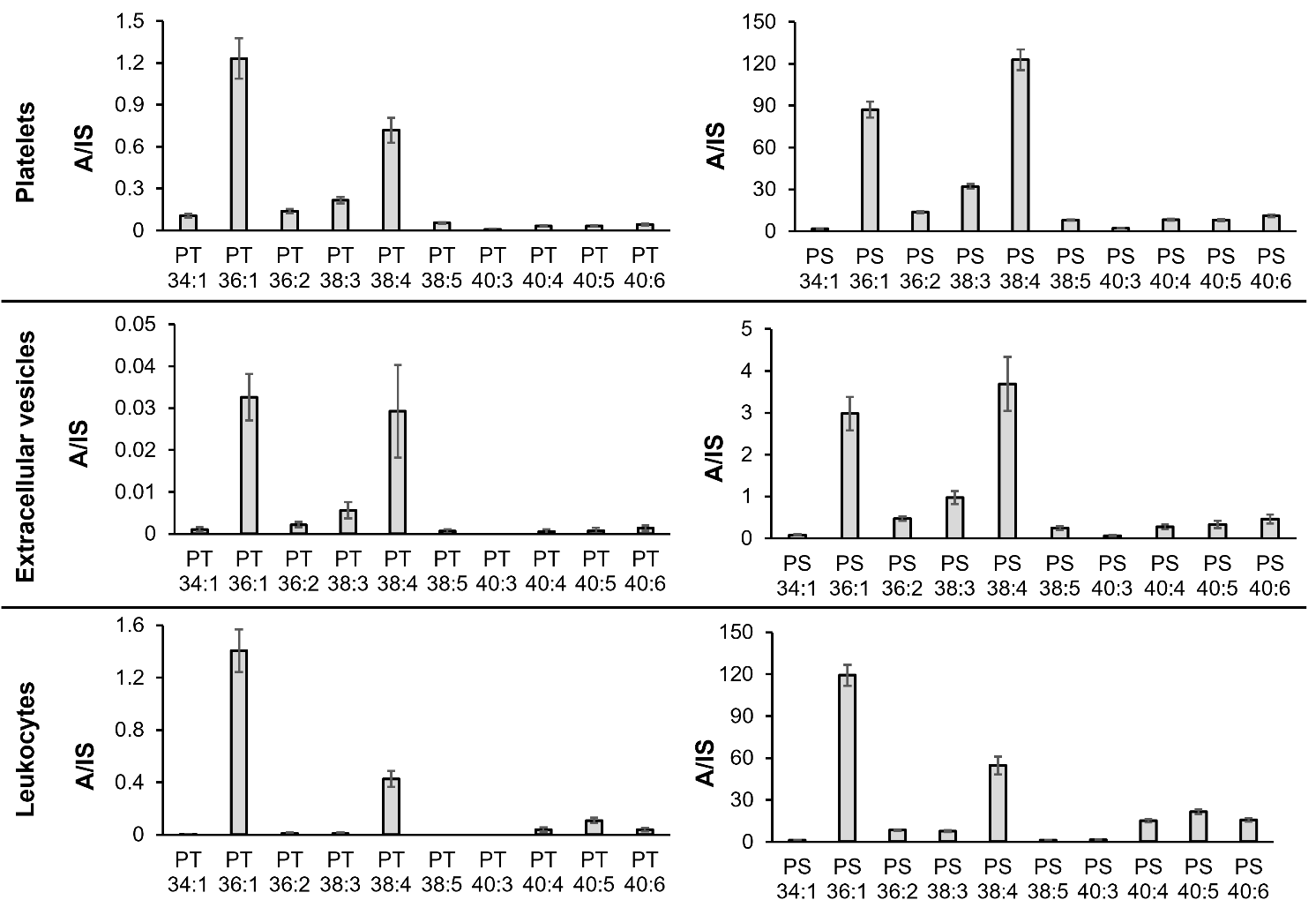


**Supplementary Figure 11. Comparison of PT and PS molecular species in human platelets, EV, and leukocytes.**
Lipid extracts of platelets, leukocytes, and EVs from healthy volunteers from the clinical cohort were analyzed for PT and PS using HILIC LC-MS/MS as described in Supplementary Methods. Analyte peak areas were integrated and ratios of analytes to internal standards (A/IS) calculated. Data are represented as mean ± SEM (n = 24).

MRM 1028.600/701.600 Da

2.0

3.0

4.0

5.0

6.0

7.0

8.0

9.0

Time, min

0

1000

2000

3000

4000

5000

6000

7000

Intensity, cps

5.26

Biotinylated PT 36:1

**Thrombin-activated**

**control**


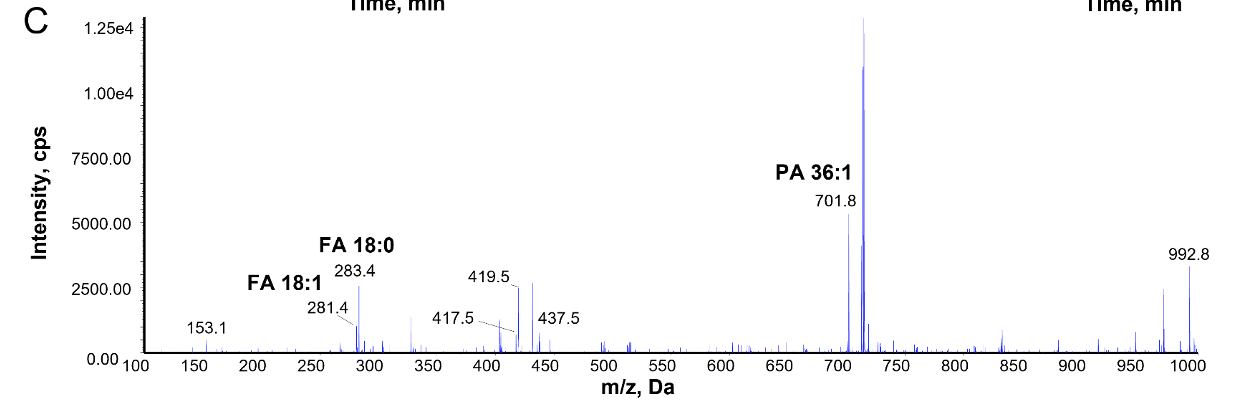


A

B

**Supplementary Figure 12. Externalized PT 36:1 is increased in platelets following thrombin activation, and MS/MS of biotinylated PT 36:1 in activated platelets.**

*Panel A. Extracted ion chromatogram of biotinylated PT 36:1 in resting (red) and thrombin-activated (blue) platelets.* Isolated platelets were treated with sulfo-NHS-biotin, then lipids were extracted and analyzed using RP-LC-MS/MS as described in Supplementary Methods (MRMs in Supplementary Table 2). Externalized PT 36:1 is increased in platelets following thrombin activation. *Panel B. MS^2^ scan of m/z 1028.6 acquired at 5.27 min, showing the expected FA peaks (m/z 281.4 and 283.4) and fragment resulting from neutral loss of biotinylated threonine head group (m/z 701.8) from biotinylated PT 36:1.* Isolated platelets were treated with NHS-biotin, then lipids were extracted and analyzed using RP-LC-MS/MS as described in Supplementary Methods.

MRM 1050.600/723.600 Da

1.0

2.0

3.0

4.0

5.0

6.0

7.0

8.0

9.0

Time, min

0.0

5000.0

1.0e4

1.5e4

2.0e4

Intensity, cps

3.87


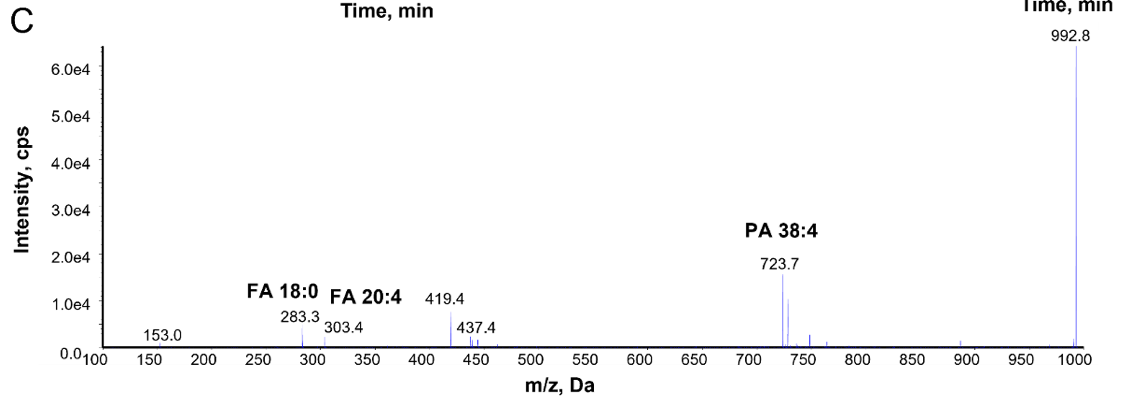


Biotinylated PT 38:4

**Thrombin-activated**

**control**

A

B

**Supplementary Figure 13. Externalized PT 38:4 level is increased in platelets following thrombin activation, and MS/MS of biotinylated PT 38:4 in activated platelets.**

*Panel A. Extracted ion chromatogram of biotinylated PT 38:4 in resting (red) and thrombin-activated (blue) platelets.* Isolated platelets were treated with sulfo-NHS-biotin, then lipids were extracted and analyzed using RP-LC-MS/MS as described in Supplementary Methods (MRMs in Supplementary Table 2). Externalized PT 38:4 is increased in platelets following thrombin activation. *Panel B. MS^2^ scan of m/z 1050.6 acquired at 3.90 min, showing the expected FA peaks (m/z 283.3 and 303.4) and fragment resulting from neutral loss of biotinylated threonine head group (m/z 723.7) from biotinylated PT 38:4.* Isolated platelets were treated with NHS-biotin, then lipids were extracted and analyzed using RP-LC-MS/MS as described in Supplementary Methods.


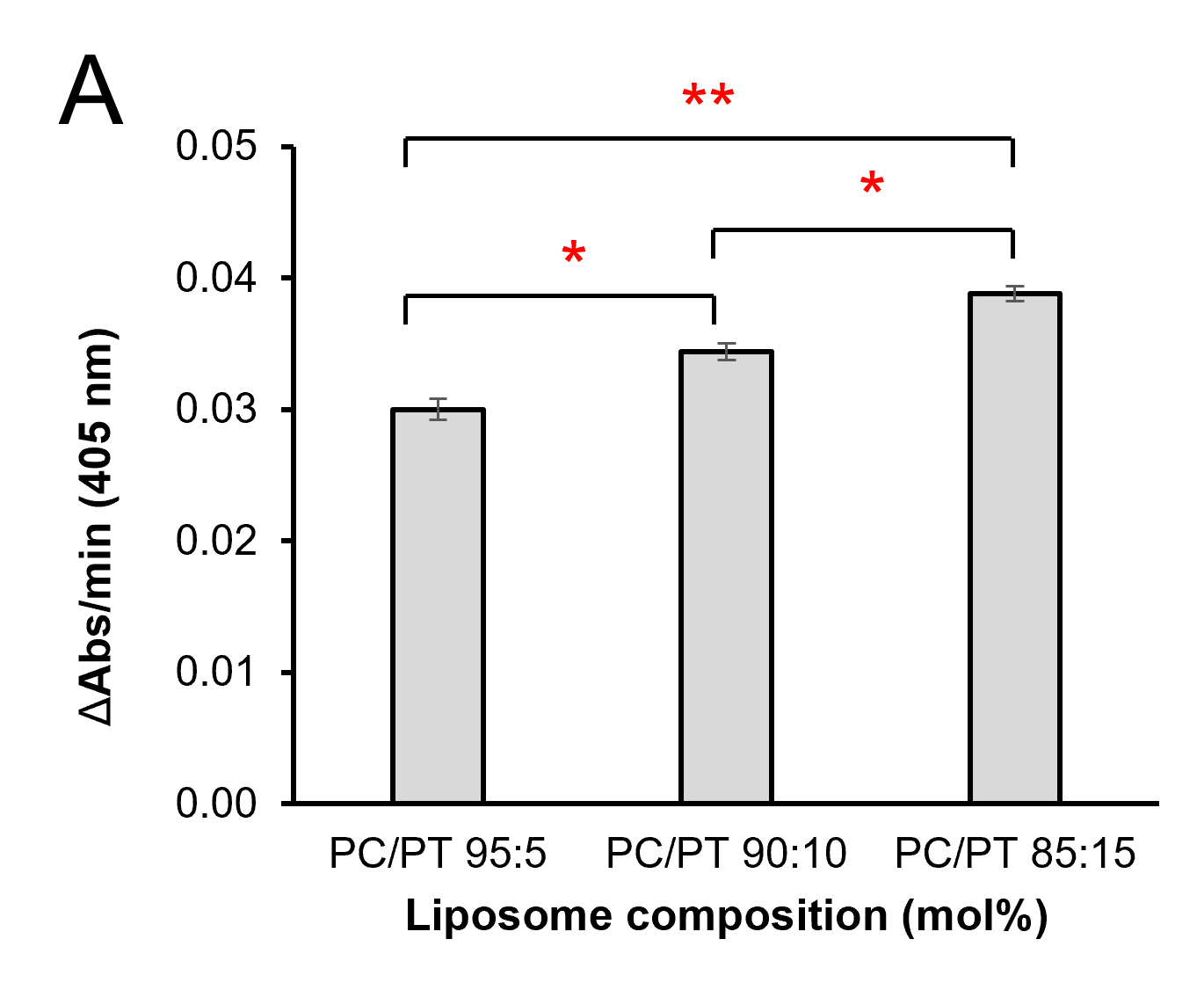


**Supplementary Figure 14. PT liposomes support prothrombinase activity *in vitro* (without PE).**

Liposomes of varying compositions were prepared by extrusion and tested using the prothrombinase assay, as described in Supplementary Methods. Prothrombinase assay: Liposomes (25 µM) were incubated with FII (200 nM), FVa (3.0 nM), FXa (10 nM), and 1 mM CaCl_2_ for 5 min. The reaction was stopped and FIIa generation was monitored for 50 min as described in Supplementary Methods (mean ± SEM, n = 3). Statistical significance was determined using one-way ANOVA and post-hoc Tukey test (*: p < 0.05, **: p < 0.01).


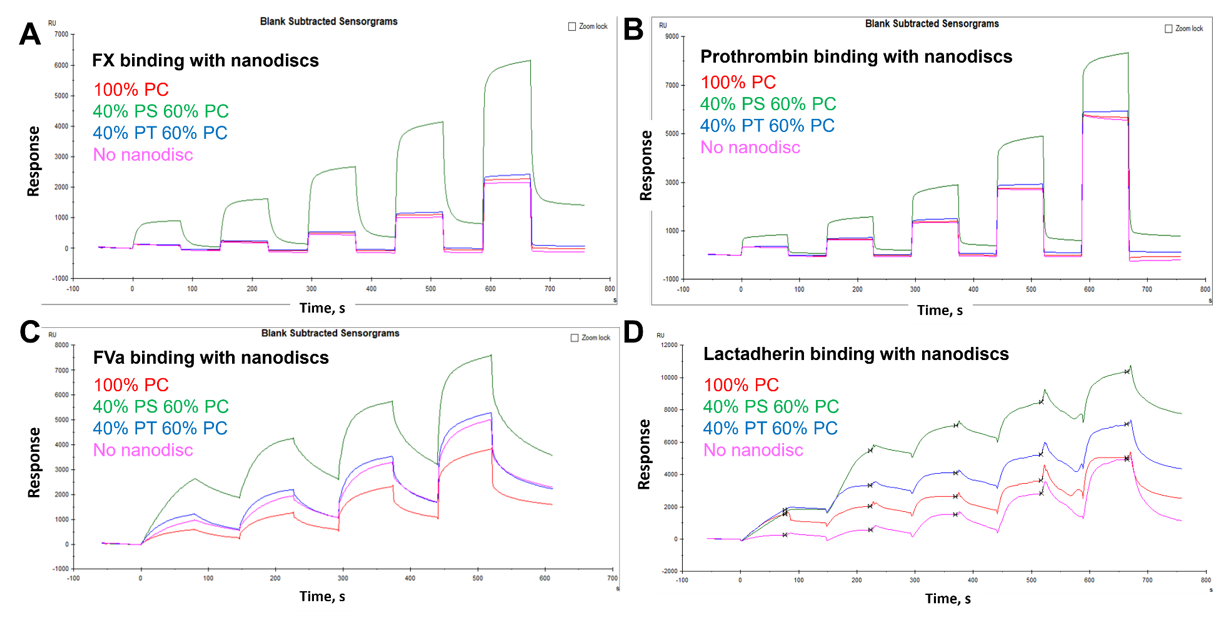


**Supplementary Figure 15. Exemplary raw sensorgrams from the SPR analysis.**

Nanodiscs were prepared and tested for their ability to bind coagulation proteins and lactadherin using SPR as described in Supplementary Methods. *Panels A,B. PT nanodiscs poorly bind FX (A) and prothrombin (B) compared to PS. Panels C,D. PT nanodiscs strongly bind FVa (C) and lactadherin (D) compared to PC.* Traces: 100% PC (red), 40% PS 60% PC (green), 40% PT 60% PC (blue), no nanodisc (pink). Note, nonspecific binding of FVa to the empty flow cell was observed (panel C).


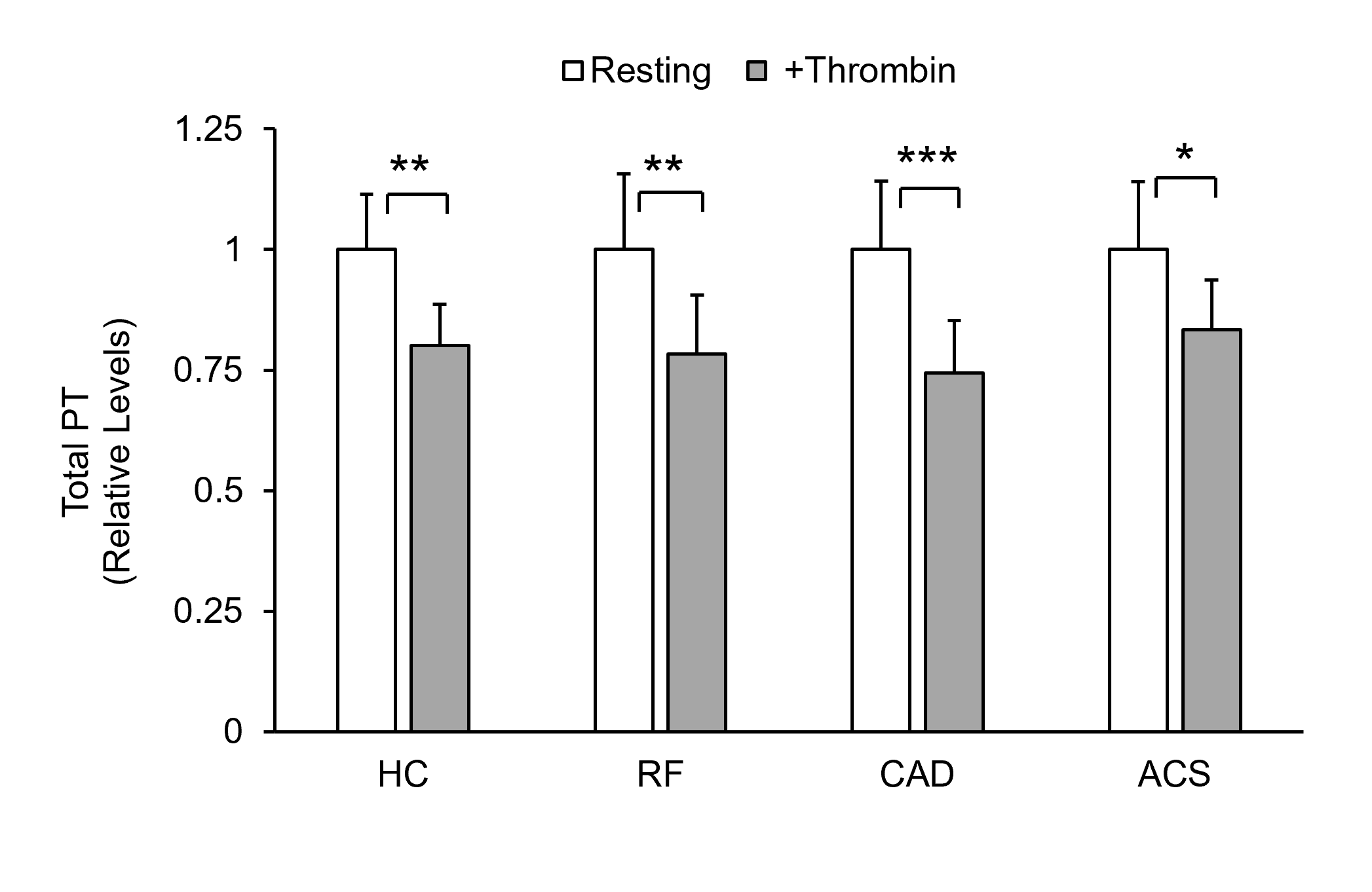


**Supplementary Figure 16. PT is similarly metabolized in response to thrombin activation in patients with arterial vascular disease.**

Lipids were analyzed for PT using HILIC LC-MS/MS, in lipid extracts of resting and thrombin-activated platelets from the clinical cohort as described in Supplementary Methods. Analyte peak areas were integrated and ratios of analytes to internal standards (A/IS) calculated. Total PT levels were calculated by summing A/IS values of individual species for the respective samples. Levels are expressed relative to PT in resting cells for each cohort group. Statistical significance was determined using paired t-tests (*: p < 0.05, **: p < 0.01, ***: p < 0.001). HC: healthy controls (n = 24), RF: risk factors with no significant coronary artery disease (n = 23), CAD: coronary artery disease but no acute coronary syndrome (n = 19), ACS: acute coronary syndrome (n = 24).


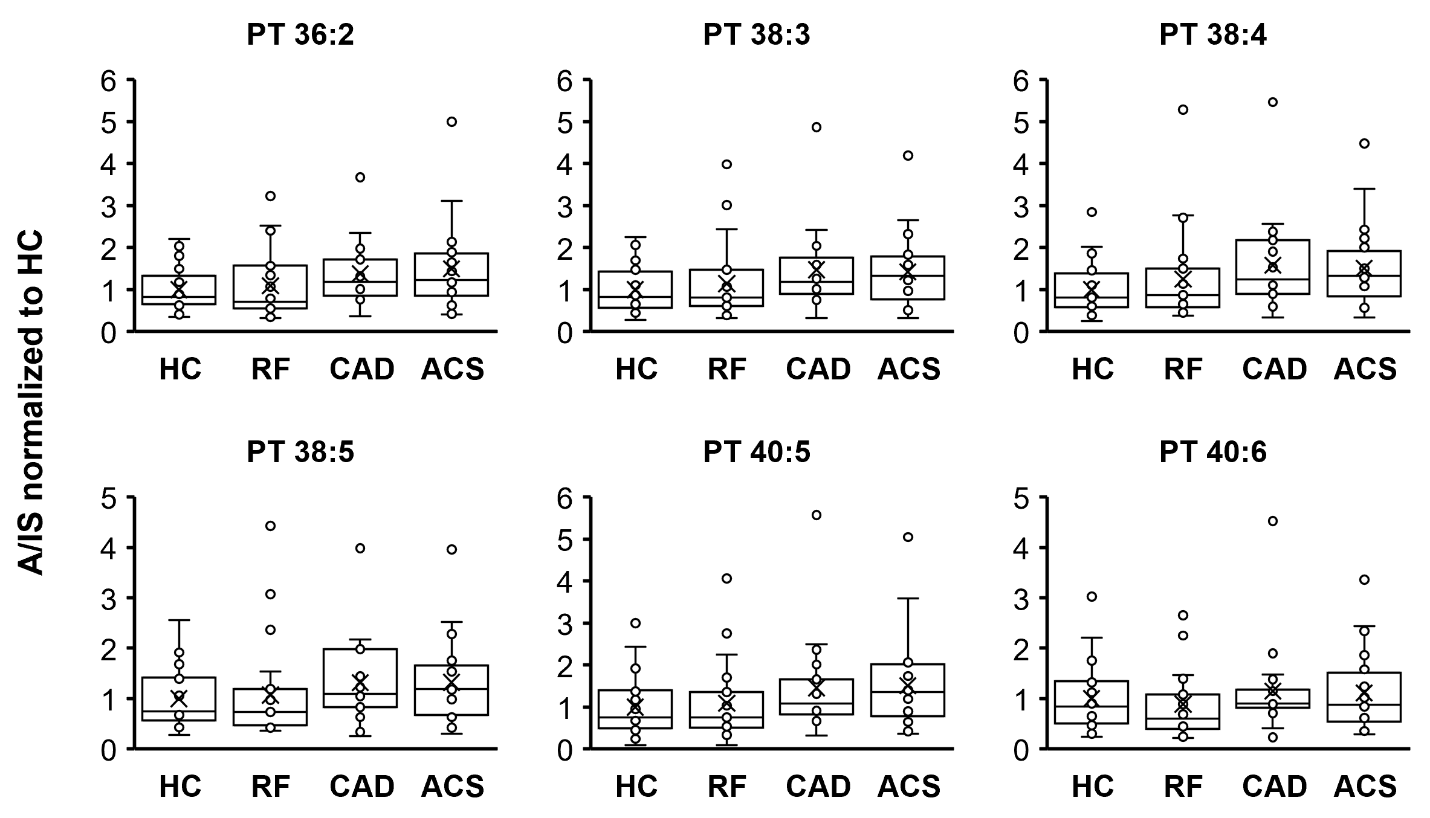


**Supplementary Figure 17. Upward trends in some PT molecular species in platelets from coronary artery disease patients compared to healthy volunteers.**

Lipid extracts from platelets (from the clinical cohort) were analyzed for PT using HILIC LC-MS/MS as described in Supplementary Methods. Analyte peak areas were integrated and ratios of analytes to internal standards (A/IS) calculated. Values were normalized to the mean of the HC group. Statistical significance was determined using Kruskal-Wallis H test (*: p < 0.05, **: p < 0.01, ***: p < 0.001). HC: healthy controls (n = 24), RF: risk factors with no significant coronary artery disease (n = 23), CAD: coronary artery disease but no acute coronary syndrome (n = 19), ACS: acute coronary syndrome (n = 24). Data are presented as box and whisker plots, in which the box edges indicate the interquartile range (IQR) with the median line inside the box. Whiskers indicate 1.5 times the IQR, and “X” represents the mean.


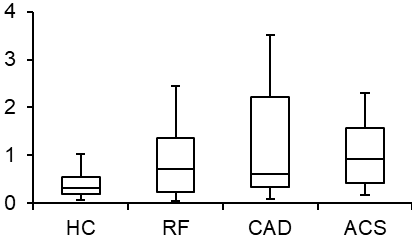

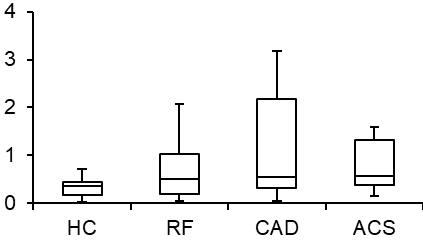

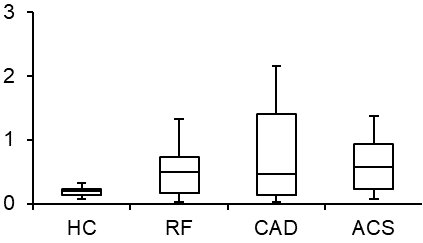

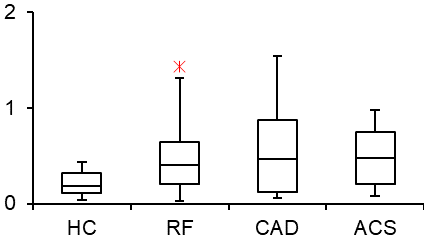


Relative levels (normalized to EV count) then outliers removed

Relative levels (normalized to EV count) then outliers removed

*

*

**

*

**

*

*

*

PT 36:1

PT 36:2

PT 38:3

PT 38:4

**Supplementary Figure 18. PT is significantly elevated in EV from patients with arterial vascular disease when samples are normalized to EV counts and outliers removed.** Lipids were analyzed for PT using HILIC LC-MS/MS, in lipid extracts from EV from a clinical cohort as described in Supplementary Methods. Analyte peak areas were integrated and ratios of analytes to internal standards (A/IS) calculated. Total PT levels were calculated by summing A/IS values of individual species for the respective samples. PT in EV was normalized using EV counts obtained using nanoparticle tracking analysis. Values were normalized to the mean of the HC group, then outliers removed. Statistical significance was determined using Kruskal-Wallis H test (*: p < 0.05, **: p < 0.01, ***: p < 0.001). HC: healthy controls (n = 18), RF: risk factors with no significant coronary artery disease (n = 18), CAD: coronary artery disease but no acute coronary syndrome (n = 17), ACS: acute coronary syndrome (n = 18). Data are presented as box and whisker plots, in which the box edges indicate the interquartile range (IQR) with the median line inside the box. Whiskers indicate 1.5 times the IQR, and “X” represents the mean.

**Supplementary Figure 19. PT levels do not vary significantly between male and female participants**. PT levels, expressed as analyte/IS were averaged for healthy controls (female 10, male 14), risk factor positive (female 11, male 12) and acute coronary syndrome patients (female 7, male 17). Mean ± SEM. Significance was tested using two-tailed Student’s t-test, and no comparisons showed significant differences.

**
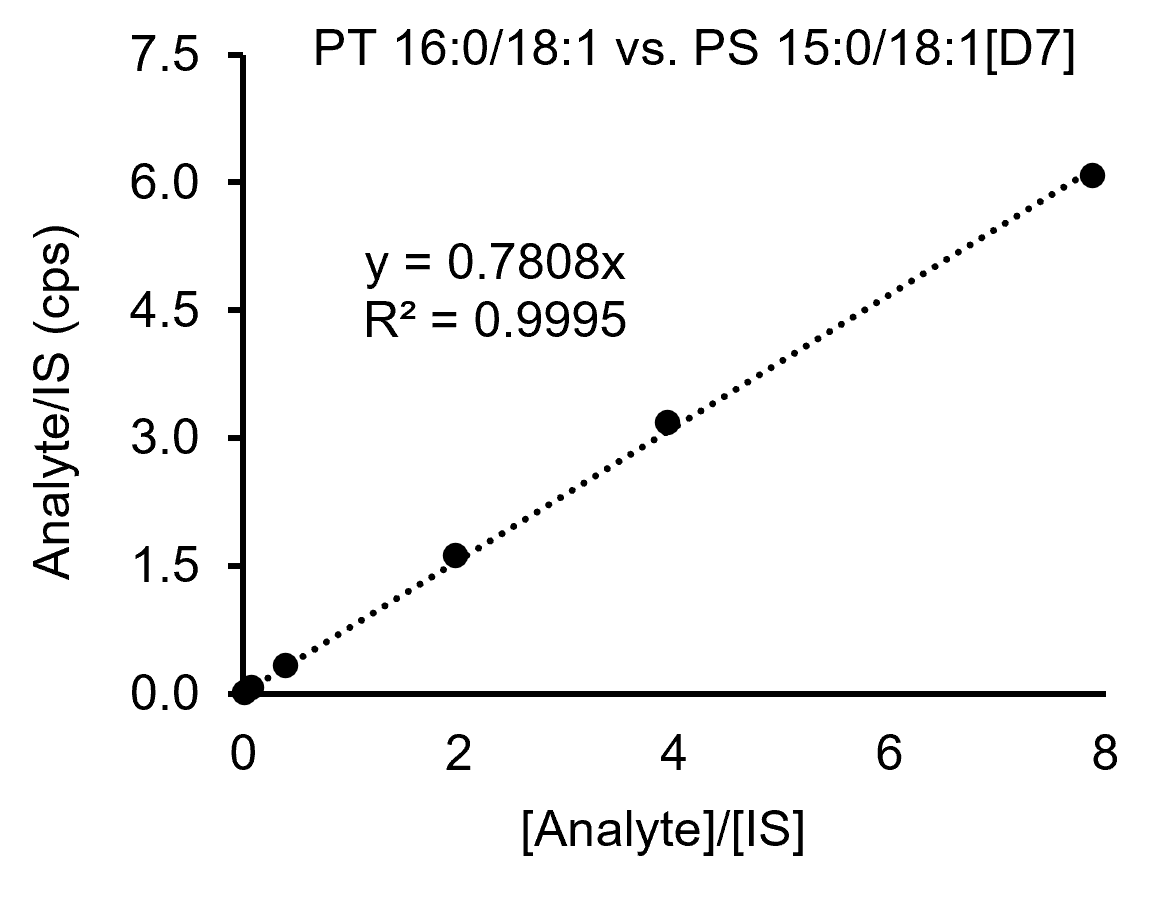
**

**
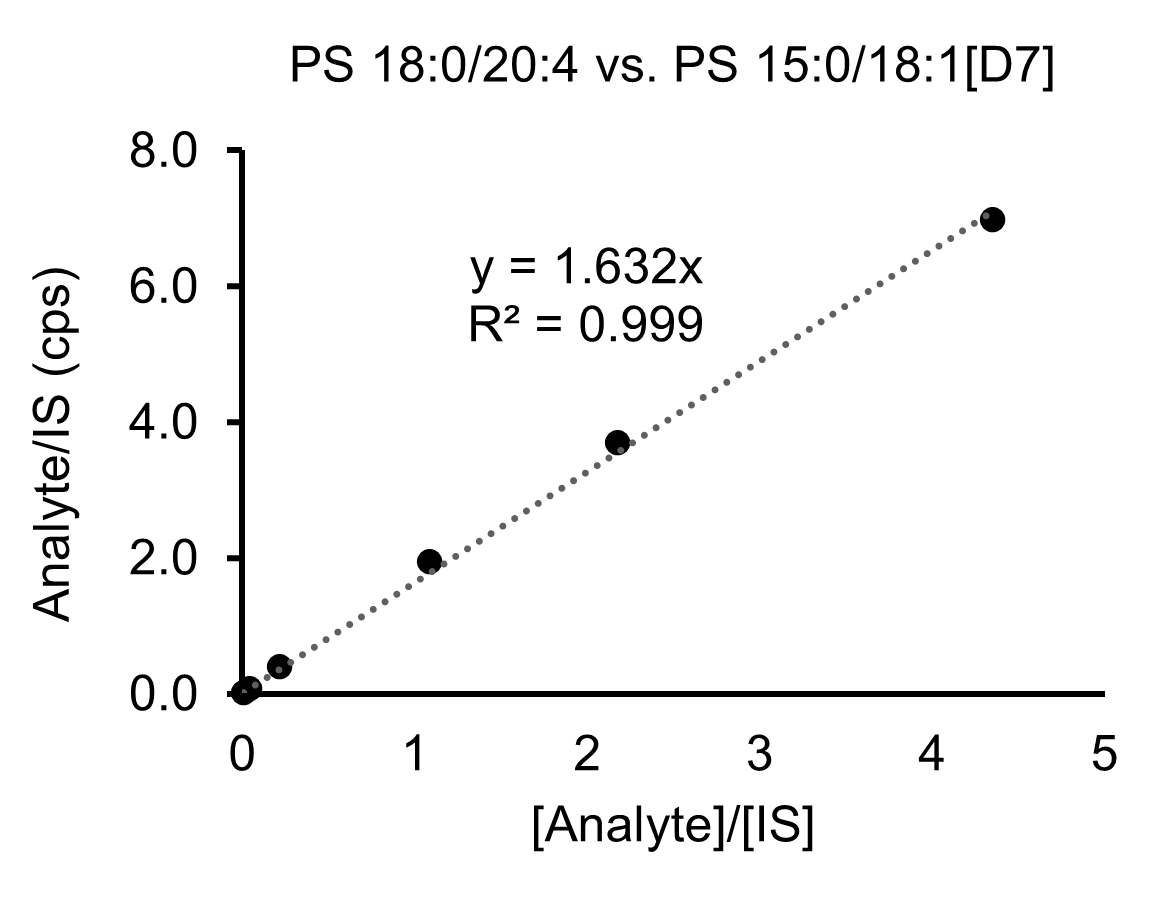
**

**Supplementary Figure 20. Six-point calibration curves of PT 16:0/18:1 vs PS 15:0/18:1[D7] (top) and PS 18:0/20:4 vs PS 15:0/18:1[D7] (bottom).**

Lipid standard solutions containing PT 16:0/18:1, PS 18:0/20:4 and PS 15:0/18:1[D7] (IS) were prepared and analyzed using HILIC LC-MS/MS as described in Supplementary Methods. Analyte peak areas were integrated and ratios of analytes to internal standards (A/IS) were calculated, then plotted against the ratio of concentrations of analyte to IS. The rate of change (slope) was calculated and used to quantify PT and PS species in whole blood lipid extracts (Figure 1 H).

**References**

1. Maskrey BH, Bermudez-Fajardo A, Morgan AH, Stewart-Jones E, Dioszeghy V, Taylor GW, et al. Activated platelets and monocytes generate four hydroxyphosphatidylethanolamines via lipoxygenase. J Biol Chem. 2007;282(28):20151-63.

2. Clark SR, Thomas CP, Hammond VJ, Aldrovandi M, Wilkinson GW, Hart KW, et al. Characterization of platelet aminophospholipid externalization reveals fatty acids as molecular determinants that regulate coagulation. Proc Natl Acad Sci U S A. 2013;110(15):5875-80.

3. Protty MB, Tyrrell VJ, Hajeyah AA, Morgan B, Li Y, Choudhury A, et al. Common anti-platelet therapies modulate procoagulant phospholipids in arterial disease. medRxiv. 2023:2022.11.03.22280948.

4. Lauder SN, Allen-Redpath K, Slatter DA, Aldrovandi M, O'Connor A, Farewell D, et al. Networks of enzymatically oxidized membrane lipids support calcium-dependent coagulation factor binding to maintain hemostasis. Sci Signal. 2017;10(507).

5. Neumann FJ, Sousa-Uva M, Ahlsson A, Alfonso F, Banning AP, Benedetto U, et al. 2018 ESC/EACTS Guidelines on myocardial revascularization. Eur Heart J. 2019;40(2):87-165.

6. Hurt L, Ashfield-Watt P, Townson J, Heslop L, Copeland L, Atkinson MD, et al. Cohort profile: HealthWise Wales. A research register and population health data platform with linkage to National Health Service data sets in Wales. BMJ Open. 2019;9(12):e031705.

7. Berckmans RJ, Lacroix R, Hau CM, Sturk A, Nieuwland R. Extracellular vesicles and coagulation in blood from healthy humans revisited. J Extracell Vesicles. 2019;8(1):1688936.

8. Coumans FAW, Brisson AR, Buzas EI, Dignat-George F, Drees EEE, El-Andaloussi S, et al. Methodological Guidelines to Study Extracellular Vesicles. Circ Res. 2017;120(10):1632-48.

9. Morgan AH, Hammond VJ, Morgan L, Thomas CP, Tallman KA, Garcia-Diaz YR, et al. Quantitative assays for esterified oxylipins generated by immune cells. Nat Protoc. 2010;5(12):1919-31.

10. Damnjanović J, Matsunaga N, Adachi M, Nakano H, Iwasaki Y. Facile Enzymatic Synthesis of Phosphatidylthreonine Using an Engineered Phospholipase D. European Journal of Lipid Science and Technology. 2018;120(6):1800089.

11. Liebisch G, Fahy E, Aoki J, Dennis EA, Durand T, Ejsing CS, et al. Update on LIPID MAPS classification, nomenclature, and shorthand notation for MS-derived lipid structures. J Lipid Res. 2020;61(12):1539-55.

12. Thomas CP, Clark SR, Hammond VJ, Aldrovandi M, Collins PW, O'Donnell VB. Identification and quantification of aminophospholipid molecular species on the surface of apoptotic and activated cells. Nat Protoc. 2014;9(1):51-63.

13. Hemker HC, Giesen P, Al Dieri R, Regnault V, de Smedt E, Wagenvoord R, et al. Calibrated automated thrombin generation measurement in clotting plasma. Pathophysiol Haemost Thromb. 2003;33(1):4-15.

14. Denisov IG, Grinkova YV, Lazarides AA, Sligar SG. Directed self-assembly of monodisperse phospholipid bilayer Nanodiscs with controlled size. J Am Chem Soc. 2004;126(11):3477-87.

15. Paul D, Morrissey JH. Stoichiometric analysis reveals a unique phosphatidylserine binding site in coagulation factor X. J Thromb Haemost. 2022;20(3):600-4.

16. Martín-Molina A, Rodríguez-Beas C, Faraudo J. Effect of Calcium and Magnesium on Phosphatidylserine Membranes: Experiments and All-Atomic Simulations. Biophysical Journal. 2012;102(9):2095-103.

17. Sinn CG, Antonietti M, Dimova R. Binding of calcium to phosphatidylcholine–phosphatidylserine membranes. Colloids and Surfaces A: Physicochemical and Engineering Aspects. 2006;282-283:410-9.
